# Peri-conceptional diet patterns and the risk of gestational diabetes mellitus in South Indian women

**DOI:** 10.1017/S1368980022001288

**Published:** 2023-04

**Authors:** Anvesha Mahendra, Sarah H Kehoe, Sarah R Crozier, Kalyanaraman Kumaran, GV Krishnaveni, Nalini Arun, Padmaja, Prakash Kini, Unaiza Taskeen, Krupa T Kombanda, Matthew Johnson, Clive Osmond, Caroline HD Fall

**Affiliations:** 1 MRC Lifecourse Epidemiology Centre, University of Southampton, Southampton SO16 6YD, UK; 2 Epidemiology Research Unit, CSI Holdsworth Memorial Hospital, Mysore, India; 3 Department of Obstetrics and Gynaecology, Bangalore Baptist Hospital, Bangalore, India; 4 Department of Obstetrics and Gynaecology, Cloudnine Hospital, Bangalore, India; 5 Previously Affiliated to Epidemiology Research Unit, CSI Holdsworth Memorial Hospital, Mysore, India; 6 School of Exercise and Nutrition, Deakin University, Geelong, Australia

**Keywords:** Peri-conceptional, Diet patterns, Prospective study, Gestational diabetes mellitus and India

## Abstract

**Objective:**

To identify peri-conceptional diet patterns among women in Bangalore and examine their associations with risk of gestational diabetes mellitus (GDM).

**Design:**

BAngalore Nutrition Gestational diabetes LifEstyle Study, started in June 2016, was a prospective observational study, in which women were recruited at 5–16 weeks’ gestation. Peri-conceptional diet was recalled at recruitment, using a validated 224-item FFQ. GDM was assessed by a 75-g oral glucose tolerance test at 24–28 weeks’ gestation, applying WHO 2013 criteria. Diet patterns were identified using principal component analysis, and diet pattern–GDM associations were examined using multivariate logistic regression, adjusting for ‘*a priori*’ confounders.

**Setting:**

Antenatal clinics of two hospitals, Bangalore, South India.

**Participants:**

Seven hundred and eighty-five pregnant women of varied socio-economic status.

**Results:**

GDM prevalence was 22 %. Three diet patterns were identified: (a) high-diversity, urban (HDU) characterised by diverse, home-cooked and processed foods was associated with older, more affluent, better-educated and urban women; (b) rice-fried snacks-chicken-sweets (RFCS), characterised by low diet diversity, was associated with younger, less-educated, and lower-income, rural and joint families; and (c) healthy, traditional vegetarian (HTV), characterised by home-cooked vegetarian and non-processed foods, was associated with less-educated, more affluent, and rural and joint families. The HDU pattern was associated with a lower GDM risk (adjusted odds ratio (aOR): 0·80/sd, 95 % CI (0·64, 0·99), *P* = 0·04) after adjusting for confounders. BMI was strongly related to GDM risk and possibly mediated diet–GDM associations.

**Conclusions:**

The findings support global recommendations to encourage women to attain a healthy pre-pregnancy BMI and increase diet diversity. Both healthy and unhealthy foods in the patterns indicate low awareness about healthy foods and a need for public education.

Gestational diabetes mellitus (GDM) is an important public health problem in India, affecting about 20% of the pregnancies^(1)^. GDM causes short- and long-term adverse health consequences for mothers and their children. Short-term problems include a higher risk of caesarean deliveries in the mother and preterm birth, macrosomia and congenital heart abnormalities for the fetus^(2)^. Long-term consequences of GDM include an increased maternal risk of type 2 diabetes mellitus (T2DM), and intra-uterine programming of insulin resistance in the offspring that increases their risk of obesity, T2DM and CVD in later life^(2)^. While diet before and during pregnancy is increasingly being recognised as an important modifiable risk factor to prevent GDM^(3)^, diet during the peri-conceptional period may be critical to prevent GDM, since, although GDM is usually detected in late pregnancy, the source of metabolic dysfunction is established much before, probably during the peri-conceptional period (six months around conception including the period of oocyte growth, fertilisation and embryogenesis)^(4,5)^. Diet patterns best capture the totality of the ‘diet’ exposure, the combinations and quantities in which foods and nutrients are consumed and overcome the limitations of single food and nutrient approaches^(3)^. Evidence linking peri-conceptional diet patterns and GDM in high-income countries in the West has found healthy or prudent patterns, with higher intakes of wholegrains, fruits and vegetables, to be associated with a lower GDM risk, and an unhealthy or ‘Western’ diet pattern with higher intakes of refined grains, fried and fast food, high sugar, red and processed meat to be associated with a higher risk^(3)^. However, definitions of ‘healthy’ patterns across the globe are heterogeneous and challenging to interpret^(6)^. Currently, a knowledge gap exists about culture-specific healthful diet patterns to prevent GDM in India. Foods in Indian and Western diets are different, although ultra-processed food intake is rising in India^(7)^. Most global interventions have focused on calorie and fat restriction to reduce gestational weight gain and macrosomia to prevent GDM^(8)^. These strategies are less applicable to India as they may decrease birth weight^(8)^ and increase the burden of GDM and T2DM^(9)^ according to the DOHaD (Developmental Origins of Health and Disease) hypothesis. The BAngalore Nutrition Gestational diabetes LifEstyle Study (BANGLES) aimed to identify peri-conceptional diet patterns in South India and examine their association with GDM risk.

## Methods

### Study location and design

**Fig. 1:**
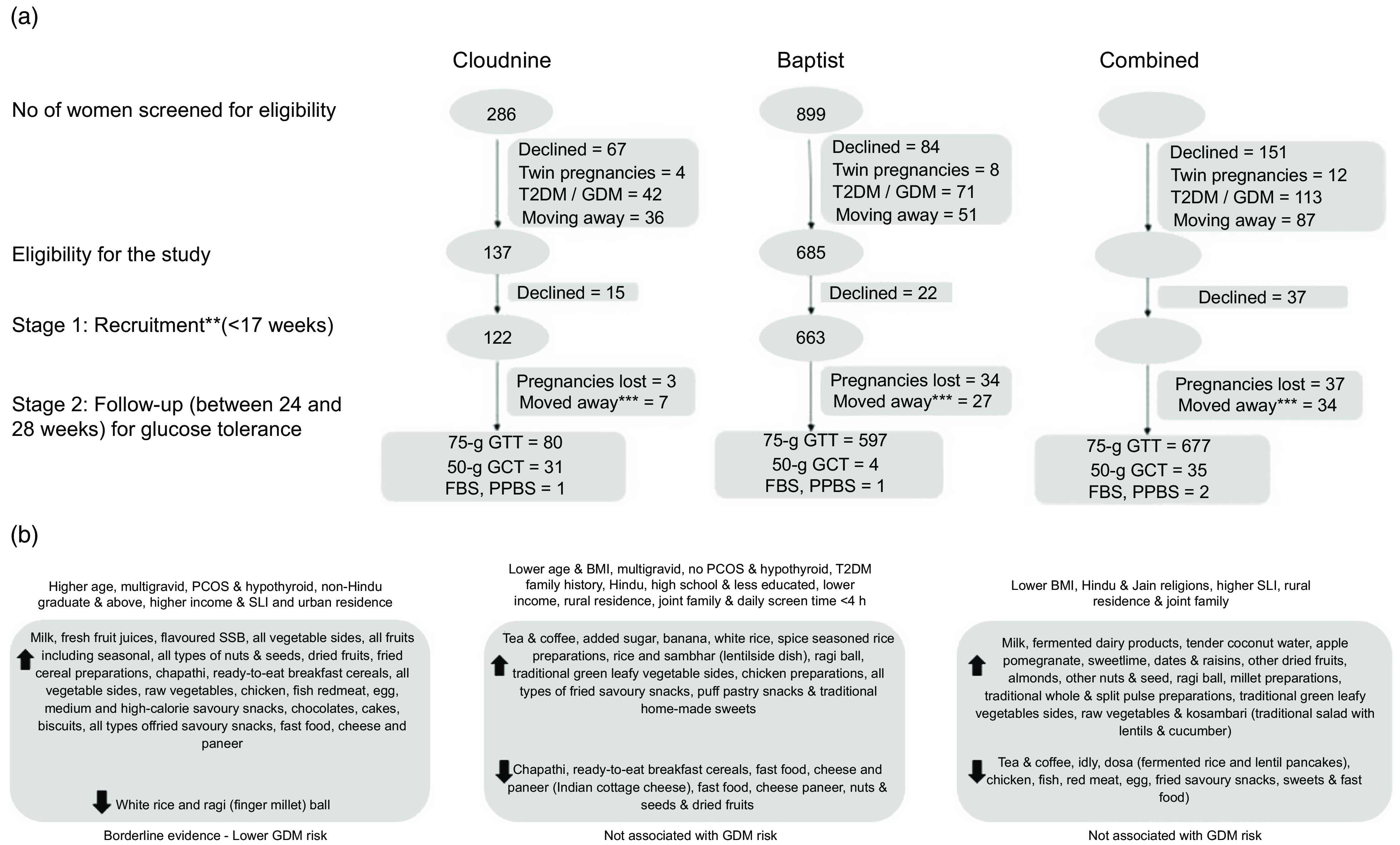
(a) BANGLES participant flow charts at Cloudnine and Baptist hospitals, and both combined. (b) Summary diagram of diet patterns and their associations with population characteristics and GDM risk. **Recruitment included data on diet, general health, socio-economic status, physical activity and blood samples; ***moved away means participants dropped out due to moving to their native place for delivery before 6 months and change of hospital. GDM, Gestational diabetes mellitus; GTT, glucose tolerance test; GCT, glucose challenge test; FBS, fasting blood sugar test; PPBS, postprandial blood sugar test; T2DM, type 2 diabetes mellitus

Bangalore, the capital of Karnataka state (South India), has a population of 8·5 million. BANGLES was a prospective observational study, in which 785 women were recruited from the antenatal clinics of two hospitals in Bangalore at 5–16 weeks of gestation (Fig. 1(a)). One hospital mainly caters to women from middle- and high-income groups, while the other caters to women from the highest income group. Inclusion criteria included (i) a singleton pregnancy, (ii) <17 weeks’ gestation, (iii) no previous history of diabetes and (iv) an intent to stay in Bangalore until at least 28 weeks’ gestation (to ensure follow-up to ascertain GDM). A consecutive recruitment process was followed in both hospitals, between June 2016 and October 2017.

### Dietary exposure

The exposure was the woman’s diet in the peri-conception period. Most women discover that they are pregnant between 2 and 4 weeks after conception; so at recruitment, we asked them to think back to what they were eating during the month before they knew they were pregnant. We recruited women between 5 and 17 weeks of pregnancy and asked them to recall their diet in the month before they know they were pregnant. Based on this, the interval from conception to recruitment ranged from 2 to 6 months. A 224-item FFQ (see online supplementary sections 1 & 2) was administered by trained nutritionists at recruitment (5–16 weeks’ gestation). The FFQ was developed from the 136-item Mysore Parthenon Study’s FFQ (MP-FFQ), used for children in Mysore^(10)^. Bangalore and Mysore in Karnataka are only 150 km apart, and the foods eaten in both places are similar. We undertook pilot work to adapt the MP-FFQ for pregnant women and to capture new-age foods, for example, ready-to-eat breakfast cereals, that were not widely consumed when the MP-FFQ was designed. Using 500 24-h recalls among pregnant women in both the study centres, frequently consumed foods were identified and food items appearing >5 times (and not already on the MP-FFQ) were added to the BANGLES FFQ. Seasonal foods were grouped separately, and the women were asked how often they consumed them when in season. The FFQ was organised into sections of similar foods types to facilitate data collection (e.g. fruits and beverages). These steps created the 224-item BANGLES FFQ (see online supplemental section 3). Dietary data were collected using an FFQ kit with familiar household vessels. The nutritionists collecting the data were fluent in local languages and used an atlas with photographs and food models. Foods that women reported eating but were not on the FFQ were recorded as ‘other foods’ in a separate section of the FFQ. These were later merged into existing FFQ items with similar nutrient composition. The BANGLES FFQ was validated using a combination of methods (see online supplemental section 4).

### Principal component analysis

Principal component analysis (PCA) is a widely used statistical technique in diet patterns research. It identifies foods that are consumed together by individuals and produces new variables (components) that are independent linear combinations of the dietary variables accounting for maximum variance^(9)^. Weekly frequency of intake of foods were selected as the input variables in order to make study findings more relatable to all settings globally (units and quantified weights of foods can be different across settings based on how a food is cooked).

#### Food group input variables for the principal component analysis

Food groups to be used as PCA input variables were constructed using a mix of *a priori and some a posteriori methods*. The a *priori method* was based on prior knowledge of foods with similar nutrient composition. For example, different types of nuts and seeds including almonds, cashew nuts, pistachios, groundnuts, walnuts, sunflower seeds, pumpkin seeds, flax seeds and chia seeds were grouped together (rationale for food grouping) (see online supplemental section 5).

The a *posteriori method* included further modifications to the *a priori* food groups to determine the final food groups as input variables for the PCA and included the following steps:

##### a) A principal component analysisof 205 individual FFQ food items

This step is to identify foods that characterise a particular diet pattern. This information may be lost when a PCA is done on pre-grouped foods. Foods with high factor loadings (factor loading >0·2) in each pattern, that stand out in a particular pattern, were retained as separate input variables in the final PCA. For example, the factor loading for chapati in pattern 1 was 0·38, and banana (yelakki) had a factor loading of 0·26 in pattern 2. These were considered discriminating foods for patterns 1 and 2 (see online supplemental section 6).

##### b) Identification of commonly consumed foods

Foods that were consumed ≥5 d/week were grouped as separate input variables, for example, rice, ragi ball, chapati and banana. This was done to prevent commonly consumed foods from diluting the discriminating power of the diet patterns. For instance, bananas were frequently consumed by most women and hence were assigned as a separate food group. Other fruits were consumed less frequently and not by all the women, so were more discriminatory. If bananas and other fruits were all grouped together, the PCA would not discriminate between the women in terms of their fruit intake because the universal consumption of bananas would mean that the factor loading for fruit would be close to 0.

##### c) Consideration of culture-based preferences of women

While interviewing the women, we formed a strong impression that they tended to make dietary choices based on their cultural inclination, preferences and affordability, more than on nutritional considerations. From a nutrition perspective, brown bread and chapati are both made of wholegrain wheat, are a good source of fibre and have similar calorie content. Keeping this in mind, Western cereal products (breads, buns and noodles) were grouped separately from the traditional Indian wheat breads (chapati and paratha).

This combination of *a priori* and *a posteri* methods created sixty-eight food groups as input variables. We used an unrotated PCA to derive the diet patterns, since some evidence indicates that rotation can lead to loss of pattern discrimination^(11,12)^. A cut-off of 0·2 was used for the factor loadings based on the interpretability of the diet patterns. Naming of diet patterns was done using a qualitative method based on the foods that were associated with the pattern^(11)^. Each component represented a particular diet pattern. Pattern scores were calculated for each woman by multiplying the weekly frequency of consumption of items from the food group with the coefficient for that food group. These values were then summed for all sixty-eight food groups to provide scores that represent the woman’s adherence to a particular pattern. Pattern *Z*-scores were then calculated with a mean (sd) of 0 (1).

### Gestational diabetes

At 24–28 weeks’ gestation, participants underwent a 2-h 75-g oral glucose tolerance test (OGTT) after an overnight fast. Venous plasma glucose concentrations at baseline, and 1 and 2 h after the glucose load, were measured using the hexokinase method. A diagnosis of GDM (based on WHO 2013 criteria) was made if any one of the following values was met or exceeded: 0 h (fasting): ≥5·1 mmol/l/92 mg/dl; 1 h: ≥10·0 mmol/l or 180 mg/dl; and 2 h: ≥8·5 mmol/l or 153 mg/dl^(13)^. The majority of women (*n* 677) completed the OGTT, but thirty-five women had, on the instruction of their obstetrician, a 50-g glucose challenge test in which GDM is diagnosed from a single blood glucose value (>140 mg/dl) 1 h after a 50-g glucose drink. All women managed as GDM by their obstetrician (139 diagnosed by 75-g OGTT and eighteen diagnosed by 50-g glucose challenge test, totally 157) were included in the analysis as cases.

### Co-variates

At recruitment, we collected information on factors that may be associated with diet and/or GDM risk, based on published literature. Height was measured to the nearest 0·1 cm using a Harpenden pocket stadiometer. Weight was measured to the nearest 100 g using a calibrated, electronic digital weighing scale. Health information included gravidity, history of polycystic ovarian syndrome or hypothyroidism and a family history of T2DM among first-degree relatives. Socio-economic status (SES) was assessed using the Standard of Living Index (SLI) from the National Family Health Survey 2 (NFHS 2)^(14)^. This creates a score based on household assets, amenities and the quality of the woman’s home, with a higher score representing higher SES. The participant’s education, occupation and family income were recorded. Education level was classified into four groups, from no schooling to postgraduate degree. Occupation was classified based on Kuppuswamy’s scale^(15)^ and the International Standard Classification of Occupations (ISCO-08)^(16)^. Physical activity was assessed using the WHO International Physical Activity Questionnaire (IPAQ) short version^(17)^. BMI was categorised into underweight (BMI < 18·5 kg/m^2^), normal weight (BMI 18·5–25·0 kg/m^2^), overweight (BMI 25·01–30·0 kg/m^2^) and obesity (BMI > 30·0 kg/m^2^). Rural or urban areas of residence were classified using their postal codes in the Census directory, in which the Government of India defines ‘urban’ areas as having a population of >5000, a density of at least 400 people per square kilometre and where the majority are engaged in non-agricultural activities^(18)^.

### Statistical analysis

#### Diet patterns

From the PCA of sixty-eight food groups, the first three components were retained in the analysis based on the break in the scree plot (see online supplemental section 7). Table 2 shows the factor loadings for the first three components without rotation. The Kaiser–Meyer–Olkin measure of sampling adequacy for the PCA was 0·73 indicating the adequacy of our sample size for PCA. The first three components explained 18·6% of variance in the sample.

#### Statistical rationale of the overall analysis

All analyses were done using SPSS version 25. Data are presented as number and percentage (for categorical variables), mean and standard deviation (for normally distributed continuous variables), and median and interquartile range (for skewed continuous variables). Three distinct peri-conceptional diet patterns were derived using the sixty-eight food group PCA, and these were the dietary exposures. Logistic regression was used to test associations between the women’s diet pattern scores and GDM as a binary outcome, firstly in an unadjusted univariate model, secondly adjusted for potential *a priori* confounders identified from the literature (the woman’s age, gravidity, history of polycystic ovarian syndrome and hypothyroidism, family history of T2DM, urban/rural residence, nuclear/joint family type, physical activity, SLI score, education level and use of supplements including folic acid, Fe, Ca, vitamin D, vitamin B_12_, and energy and protein supplements) and thirdly adjusted for all the above plus BMI. An additional sensitivity analysis was carried out among women with a 75-g OGTT only, because a 75-g OGTT is considered as a more robust GDM screening method^(13)^, and to explore if excluding cases diagnosed by other methods would change any observed findings. All *P* values presented were two-tailed, and *P* < 0·05 was considered statistically significant.

## Results

### Study population characteristics

A total of 785 women took part in the study, and 714 were assessed for GDM. GDM was diagnosed in 157 (21·9 %). Table 1 describes the women’s characteristics. Overall, their mean age was 26·7 years and median BMI 23·8 kg/m^2^, with one-third overweight or obese (BMI > 25 kg/m^2^). Sixty-seven per cent of participants were primigravid, 33 % reported a family history of T2DM, 68 % were educated to at least graduate level and 71 % were homemakers. Median monthly family income was INR 30 000 (approximately GBP 300). Sixty-eight per cent lived in urban areas. Many women had low levels of physical activity – nearly half reported not doing any weekly light walking. Women who developed GDM were older, had a higher BMI, and were more likely to be urban, multigravid, living in a nuclear household, and to have a family history of T2DM. Of all the characteristics, higher BMI was the strongest predictor of GDM.

**Table 1:** Population characteristics among GDM and non-GDM women (*n* 714†)

Population characteristics (continuous variables)	Mean/median	sd/IQR	GDM *n* 157		Non-GDM *n* 557		*P* ‡
			Mean/median	sd/IQR	Mean/median	sd/IQR	
Age (years)§	26·7	4·3	27·6	4·6	26·4	4·1	0·005
Early pregnancy weight (kg)‖	57·0	50·0, 66·0	61·8	53·6, 71·4	56·1	49·0, 64·5	<0·001
BMI based on early pregnancy weight (kg/m^2^)‖	23·8	20·4, 26·5	25·0	22·1, 28·2	22·0	19·9, 25·9	<0·001
Total family monthly income† (INR)‖	30 000·0	15 000·0, 60 000·0	33 500·0	18 000·0, 68 750·0	30 000·0	15 000·0, 60 000·0	0·68
Standard of Living Index score§	38·0	33·0, 43·0	38·0	33·0, 42·0	38·0	33·0, 42·0	0·87

Population characteristics (categorical variables)	*n*	%	GDM *n* 157		Non-GDM *n* 557		*P* ‡
			*n*	%	*n*	%	
Categories of BMI†							
<18·50 (underweight) (reference)	67	9·4	6	3·9	61	11·3	Ref
18·5–24·9 (normal)	379	53·1	71	46·1	308	57·2	0·05
25–29·9 (overweight)	186	26·1	57	37·0	129	24·0	0·001
>30 (obese)	60	8·4	20	13·0	40	7·4	0·001
Number of previous pregnancies							
None	378	52·9	72	45·9	306	54·9	Ref
At least one	336	47·1	85	54·1	251	45·1	0·04
Family history of type 2 diabetes mellitus among first-degree relatives*							
No	481	67·4	93	59·2	388	69·7	Ref
Yes	233	32·6	64	40·8	169	30·3	0·01
History of PCOS†							
No	638	89·5	138	88·5	500	89·8	Ref
Yes	75	10·5	18	11·5	57	10·2	0·64
Hypothyroidism diagnosis†							
No	583	81·9	122	78·2	461	82·9	Ref
Yes	129	18·1	34	21·8	95	17·1	0·18
Physical activity							
No light walking	322	45·1	72	46·5	250	45·1	Ref
Light walking (1–150 min/week)	247	34·6	54	34·8	193	34·8	0·88
Light walking (>150 min/week)	145	20·3	29	18·7	111	20·0	0·69
Religion†							
Hindu	556	77·9	119	75·8	437	78·5	Ref
Christian	101	14·1	26	16·6	75	13·5	0·33
Muslim	39	5·5	9	5·7	30	5·4	0·81
Jain	9	1·3	1	0·6	8	1·4	0·47
Participant’s education level							
High school or less	105	14·7	22	14·0	83	14·9	Ref
Pre-university college	123	17·2	24	15·3	99	17·8	0·79
Graduate	285	39·9	68	43·3	217	39·0	0·55
Postgraduate or professional degree	201	28·2	43	27·4	158	28·4	0·93
Participant’s current occupation							
High-level professional	69	8·8	8	5·1	55	9·9	Ref
Mid-level professional	71	9·0	18	11·5	44	7·9	0·03
Highly skilled or technically trained occupation	57	7·3	13	8·3	41	7·4	0·12
Skilled occupation	18	2·3	1	0·6	15	2·7	0·50
Semi-skilled occupation	9	1·1	3	1·9	6	1·1	0·12
Unskilled occupation	4	0·5	0	0·0	4	0·7	0·10
Homemaker	557	71·0	111	70·7	382	68·6	0·08
Student	69	8·8	3	1·9	10	1·8	0·34
Residence							
Urban	487	68·2	118	75·6	369	67·7	Ref
Rural	214	30·0	38	24·4	176	32·3	0·05
Family type							
Nuclear	557	78·0	94	59·9	287	51·5	Ref
Joint	157	22·0	63	40·1	94	48·5	0·06

GDM, gestational diabetes mellitus; INR, Indian National Rupees; PCOS, polycystic ovarian syndrome; IQR, inter quartile range.

* First-degree relatives refer to father, mother and siblings.

† Missing data: BMI = 23, PCOS = 1, hypothyroidism = 2, religion = 9, participant’s husband’s occupation = 14, total family monthly income = 148, area of residence = 13.

‡ *P* values reported from univariate logistic regression analysis of population characteristics as predictors of GDM.

§ Mean (SD) reported.

‖ Median (IQR) reported.

### Peri-conceptional diet patterns

#### Constituents of the three diet patterns

Table 2 shows the three diet patterns identified: (i) the high-diversity, urban (HDU) pattern, characterised by consumption of a high-diversity diet (many foods in every food group having higher positive factor loadings) of expensive, home-cooked, processed, healthy and unhealthy foods including whole grains, fruits, vegetables, dairy products, nuts, seeds, egg, poultry, meat, fast food and sweets. The main staple was chapati (whole-wheat pancake); (ii) the rice-fried snacks-chicken-sugar pattern (RFCS), characterised by low diet diversity (labelled low diversity due to fewer foods consumed in this pattern), with frequent intakes of a white rice dishes (the staple cereal for this pattern), chicken dishes, shop-bought and home-made fried snacks, and a high sugar intake in tea, coffee and sweets. The main fruit was banana; (iii) the healthy traditional vegetarian pattern (HTV), characterised by frequent intake of vegetarian, healthy, home-cooked, traditional dishes including millets (ragi ball, rotis made of finger millet/ragi, sorghum/jowar and pearl millet/bajra) as the staple cereal, fermented dairy products (curds and buttermilk), tender coconut water and a moderate variety of fruits (apple, pomegranate and sweet lime), pulses and legumes, cooked greens, raw vegetables including traditional salads (kosambari), dried fruits and nuts. This pattern was devoid of any non-vegetarian and unhealthy foods such as processed fried and fast foods and sweets. The median frequency of intake of key foods characteristic of each diet pattern showed convincing trends across quintiles of the diet pattern scores (see online supplemental section 8).

**Table 2:**
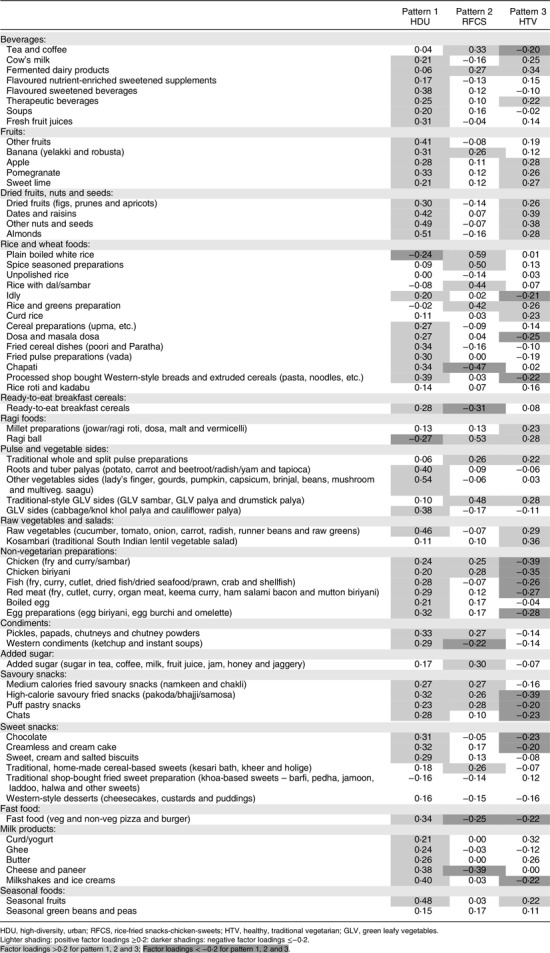
Principal component analysis of sixty-eight food groups showing factor loadings of the first three diet patterns (*n* 785)

#### Validation of the three diet patterns

There are no ‘rules’ by which to construct food groups in PCA; it is to some extent subjective to a researcher’s perspectives^(11,12)^. It has been argued that food grouping in PCA is arbitrary, and that this may affect the results^(11)^. We therefore compared the components obtained from all 205 foods with those obtained from the reduced number of sixty-eight food groups. There were strong correlations between the first three components or diet patterns derived by both methods, suggesting that despite the subjectivity of the methods, this did not lead to much difference in the results (see online supplemental section 9).

### Associations between population characteristics and women’s diet patterns scores

The HDU pattern was associated with higher diet diversity and with women being educated to graduate level or above, having higher incomes and SLI, and urban residence. Also, they were older, were more likely to be multigravid and have a diagnosis of polycystic ovarian syndrome and/or hypothyroidism and a family history of T2DM. The RFCS pattern was associated with lower education and income and being Hindu, educated to the level of high school and less, living in a joint and rural household and having a low daily screen time (<4 h). The HTV pattern was associated with a lower BMI, the Hindu and Jain religions, a higher SLI, rural residence and living in a joint family (Table 3). Higher SES was associated with higher scores for the high diet diversity patterns (HDU and HTV), and lower SES was associated with higher scores for the low diet diversity (RFCS) pattern.

**Table 3:**
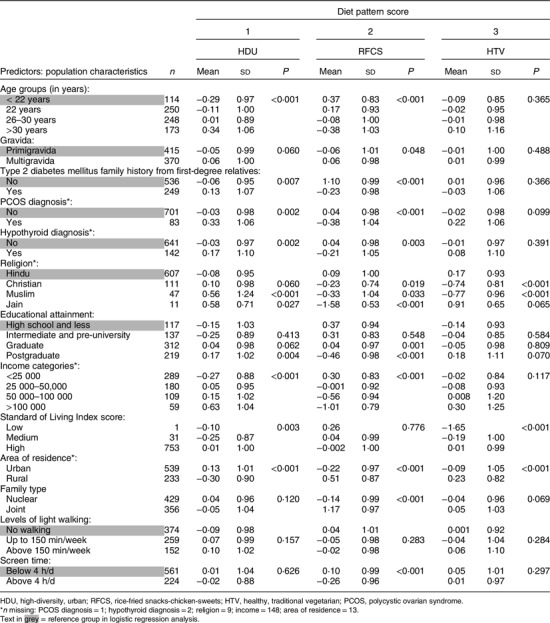
Univariate regression analysis of population characteristics as predictors of women’s diet pattern scores (*n* 785*)

### Associations between diet patterns and BMI

Higher scores on the HDU (*β*: −0·42, 95 % CI (−0·806, −0·038), *P* = 0·03), RFCS (*β*: −0·62, 95 % CI (−1·04, −0·20), *P* = 0·004) and HTV (*β*: −0·64, 95 % CI (−1·01, −0·28), *P* = 0·001) diet patterns were associated with a lower BMI, after adjusting for *a priori* confounders.

### Association between diet patterns and gestational diabetes mellitus

Table 4 shows the three regression models. Model 1 was the univariate model. Model 2 was adjusted for all the ‘*a priori*’ confounders based on previous literature, with and without adjustment for BMI, that included all 714 women who completed glucose tolerance (thirty-five women who had a glucose challenge test and two women with fasting and postprandial blood glucose tests). Model 3 was a sensitivity analysis consisting of the 677 women who had 75-g OGTT results only. Higher scores of the HDU pattern were associated with a significantly lower risk of GDM (adjusted odds ratio (aOR): 0·80, 95 % CI (0·64, 0·99), *P* = 0·04 in model 2). When BMI was added to all the models, the HDU pattern–GDM association weakened, suggesting that the association may be partly mediated by BMI. There was no evidence that the other two diet patterns were related to GDM risk.

**Table 4:** Association between women’s diet pattern scores and GDM

DP scores (sd)	GDM: 0 = no, 1 = yes														
	Univariate model*			Multivariate model†						Multivariate model‡					
	–			Model without BMI			Model with BMI			Model without BMI			Model with BMI		
	*n* 714			*n* 700			*n* 677			*n* 664			*n* 641		
	OR	95 % CI	*P*	OR	95 % CI	*P*	OR	95 % CI	*P*	OR	95 % CI	*P*	OR	95 % CI	*P*
HDU	0·90	0·75, 1·08	0·29	0·80	0·64, 0·99	0·04	0·84	0·67, 1·05	0·12	0·65	0·65, 1·01	0·04	0·84	0·67, 1·05	0·12
RFCS	0·87	0·72, 1·05	0·15	0·89	0·71, 1·11	0·32	0·96	0·76, 1·21	0·75	0·76	0·76, 1·18	0·63	1·01	0·81, 1·27	0·92
HTV	0·93	0·77, 1·12	0·45	0·95	0·78, 1·16	0·65	0·98	0·80, 1·21	0·90	0·80	0·80, 1·20	0·83	1·01	0·82, 1·25	0·92

GDM, gestational diabetes mellitus; HDU, high-diversity, urban; RFCS, rice-fried snacks-chicken-sweets; HTV, healthy, traditional vegetarian; T2DM, type 2 diabetes mellitus.

* Unadjusted model.

† Model adjusted for *a priori* confounders including woman’s age, gravidity, polycystic ovarian syndrome status (no/yes), area of residence (urban/rural), family type (nuclear and joint), family history of T2DM (no/yes), use of nutrient supplements (Ca, vitamin D, vitamin B_12_, Fe, folic acid and energy and protein supplements) and physical activity (weekly light walking), and socio-economic status measures (Standard of Living Index score, education and occupation levels).

‡ Model for sensitivity analysis, limited to women who had a full oral glucose tolerance test, adjusted for the data-driven confounders including woman’s age, family history of T2DM (no/yes), area of residence (urban/rural) and family type (nuclear/joint).

## Discussion

### Study summary

Three peri-conceptional diet patterns were derived using PCA, namely the HDU pattern, the RFCS pattern and the HTV pattern. Pattern 3 comprised mainly healthy foods, while patterns 1 and 2 contained both ‘healthy’ and ‘unhealthy’ foods. The HDU pattern was associated with a lower GDM risk after adjusting for confounders. BMI was the strongest risk factor for GDM and adjusting for BMI attenuated associations between all diet patterns and GDM.

### Characteristic foods of the diet patterns and their associations with the socio-economic factors

The HDU pattern included a diversity of vegetarian and non-vegetarian home-cooked traditional dishes with fruits and vegetables as well as urban processed shop-bought foods, available in urban Bangalore. This pattern showed higher intakes of milk, tender coconut water, soups and fresh fruit and sugarcane and juices as well as flavoured sweetened beverages. Various fruits including banana, apple, pomegranate, sweet lime, chickoo, green grapes, guava, papaya, pear, pineapple, mixed-fruit salad and avocado, and nuts, seeds and dried fruits from almonds to flax seeds to figs were consumed. Dairy products and seasonal foods including mango and strawberry were consumed. Whole-wheat chapati was the staple cereal-based food and traditional breakfast dishes (upma and dosa), and Western-style wheat products (bun, bread and noodles) and ready-to-eat breakfast cereals (cornflakes and muesli) were consumed. Various cooked vegetable sides including roots, tubers, lady’s finger and gourds, green leafy sides including drumstick, cabbage and cauliflower and raw vegetables and greens from cucumber to lettuce were consumed. Poultry, fish, meat, seafood and egg also featured here. Pickles, tomato ketchup, fried snacks from chips, samosa and Manchurian (batter-fried cauliflower) and fast food including pizzas and burgers and chocolates, cakes and biscuits were eaten. This pattern was associated with older, more affluent, more educated and urban women. Expensive foods including nuts (INR 1500/GBP 14·68 per kilogram), a diversity of seasonal and non-seasonal fruits (e.g. pomegranates cost INR 128/GBP 1·25 per kilogram and ripe mangoes cost around INR 100/GBP 0·99/fruit in all seasons)^(19)^ and chicken and mutton costing INR 350 and 500 (GBP 3·43 & 4·89) per kilogram, respectively^(20)^, were consumed (Fig. 1(b)).

The RFCS pattern was characterised by low diet diversity, with a focus on rice, fried snacks, chicken dishes and sweets. Beverages included tea, coffee and buttermilk, and the main fruit eaten was banana. More frequent intakes of rice dishes with lentils, greens and spices, traditional dishes of greens and chicken (biriyani and curry), pickles, fried snacks, home-made sweets and added sugar in tea and coffee were observed. This pattern was associated with younger, thinner, less-educated women from lower-income, rural and joint families. Women adhering to this pattern were younger, thinner, multigravid, and without polycystic ovarian syndrome, thyroid problems or a family history of T2DM. Non-expensive foods including bananas, ragi ball, white rice and fried savoury snacks in the pattern resonate with the lower SES. In government-led subsidised ration shops, low-quality rice is as cheap as INR 2/GBP 0·01/kg^(21)^ sufficient for a family of four for a week. If a family has mostly rice, dal, basic vegetables (including potato, tomato and onion) and bananas, similar to this diet pattern, their monthly household food budget would be less than INR 5000/GBP 48·5.

The HTV pattern was characterised by a diverse intake of healthy traditional and vegetarian home-cooked foods. More frequent intakes of milk, fermented dairy products (buttermilk), tender coconut water, a moderate variety of fruits (apple, pomegranate and sweet lime) and dried fruits (figs, dates and raisins) and nuts and seeds were observed. Staple cereal preparations included millets such as ragi ball, whole pulse and lentil preparations, rice with greens and curds, raw vegetables, kosambari, curd, and ghee (clarified butter) and seasonal fruits were frequently consumed. Lower consumption of Western-type bread, non-vegetarian dishes, fried and fast food and sweets was noted. This pattern could be interpreted as the ‘Vegetarian-Indian equivalent’ of the Mediterranean diet pattern in a Western setting. Nutrient-wise, this pattern was high in diet diversity and nutrients including fibre, vitamins, minerals and antioxidants; food-wise, the pattern was rich in wholegrains, legumes and lentils, nuts, seeds, fresh fruit and dried fruit, raw vegetables, yogurt, milk and green leafy vegetables. This pattern was associated with a lower BMI, the Hindu and Jain religions, a higher SLI, rural residence and living in a joint family.

### Comparing diet pattern–gestational diabetes mellitus findings with the literature

Globally, the results of diet pattern studies are inconsistent across different regions due to the heterogeneity of diets. A reason for most Asian and Indian studies not finding the clearly distinct healthy and unhealthy diet patterns seen in Western studies could be due to low awareness among people in Asia/India about healthy and unhealthy foods^(6,10)^. The Growing Up in the Singapore Towards Healthy Outcomes study found that a ‘vegetable-fruit-rice’-based diet pattern was associated with higher fasting blood sugar levels and a ‘seafood-noodle-based diet’ was inversely associated with GDM risk^(22)^. These results were attributed to noodles having a lower glycaemic index than white rice^(22)^. Similarly, in our study, women who adhered to the HDU pattern consumed chapati more frequently than white rice. Studies in other upper-middle-income countries such as Brazil^(23)^ found three diet patterns, the traditional, Western and mixed patterns, but found no difference in GDM risk. Similarly, Sedaghat *et al.* in Iran identified prudent and Western diet patterns but found no association between the prudent pattern and GDM risk^(24)^. Global characteristics of healthy diet pattern studies are higher consumption of whole grains, fruits and vegetables and nuts and seeds and lower consumption of refined grains, red and processed meat, fried food such as fries and fast food such as pizza and burgers, sweets, desserts and sugar-sweetened beverages^(3,5,6)^.

### Potential mechanisms of the high-diversity, urban diet pattern–gestational diabetes mellitus association

Women with GDM have pre-pregnancy *β*-cell dysfunction and insulin resistance^(1)^; and adherence to healthful dietary components in the peri-conceptional period may be helpful in lowering GDM risk, by abating some metabolic pre-dispositions to GDM^(25)^. The HDU pattern included a high intake of a variety of wholegrains, fruit, vegetables, greens, nuts and seeds, dried fruits and dairy products. It is biologically plausible that eating these foods leads to ingestion of key macro and micronutrients including fibre, polyphenols, vitamins, minerals and unsaturated fatty acids, thus enabling a gradual and controlled blood glucose rise and lesser adiposity^(3)^. Women adhering to the HDU pattern possibly swapped white rice to whole-wheat chapati. White rice has a high glycaemic index, and its intake in Asian populations has been associated with a higher T2DM risk^(25)^. Unhealthy food intake including biscuits and cakes may be limited, for instance, women in the highest quintile of the HDU diet pattern had a median frequency of intake of biscuits and cakes of two per week. While meat intake is rising in India^(26)^, its annual intake is still low (5 kg/capita) compared to developed countries (120 kg/capita)^(27)^. No Indian study has linked meat intake to higher T2DM or GDM risk, and unprocessed meat has not been associated with a higher GDM risk globally. Unexpectedly, the other diet patterns were not associated with GDM risk, and the HTV pattern was not associated with a lower GDM risk. Similar to our findings, other global studies have also not found any association between traditional/prudent diet patterns that are healthy and GDM risk^(23,24)^. Diet patterns studies in India have not captured clearly healthy/prudent diet pattern *v*. unhealthy diet patterns, where modern *v*. traditional diet patterns have been reported and religion/SES are the main determinants of diet^(10)^.

### BMI as a mediator

Lower scores on the HDU diet pattern were associated with a higher BMI, and adding BMI to the regression analysis relating the HDU pattern to GDM diminished the association, suggesting that it may be partly mediated by BMI.

### Study strengths and limitations

BANGLES is the first Indian study investigating diet pattern–GDM associations. The cohort design incorporated temporality with dietary data collection during early pregnancy, before GDM diagnosis. Excluding women with any type of prior diabetes or GDM ensured that the participants had not changed their diets for health reasons before the diet assessment. Trained nutritionists collected dietary data face to face and using a validated FFQ which minimised potential errors in exposure measurement^(28)^. Recall bias was minimised as much as possible using tools (food atlas and food models) to make the recall process easier for the participants. All the FFQ were administered in a standardised manner, and recall bias is less likely to affect the study as neither the participant nor interviewer was aware of the outcome. Hence, we expect the recall bias would be random and distributed equally across the GDM and non-GDM women. Using frequency of intake as a dietary exposure over quantified amounts makes the findings generalisable to settings elsewhere in the world. Statistical modelling addressed confounding by adjusting for a range of *a priori* confounders. A limitation of the study was that women were recruited in early pregnancy and were asked to recall their diet around the time of conception. Furthermore, the interval between conception and recruitment varied between women (from 2 to 6 months). We acknowledge that this could introduce recall bias. It was not feasible to recruit women pre-conceptionally, as large numbers of women would be required in order to obtain sufficient pregnancies. Another limitation was that our sample did not include the very poorest sections of society (annual income of <INR 10 000/GBP 97), but despite this there was a wide variation in SES. Gestational weight gain could not be measured in the study.

## Conclusion and public health implications

BANGLES identified three characteristic peri-conceptional diet patterns among women from rural and urban Bangalore. The HDU pattern was associated with lower GDM risk. One single healthy diet pattern may not be relevant in India^(4)^ where food, culture and attitudes are very diverse across the country. Future work could use pooled data to study diet patterns across India, which may enable future researchers and policymakers to design culturally relevant food-based policies and interventions to prevent GDM.

Our findings have potential public health policy implications. The fact that higher BMI was the strongest risk factor for GDM means that future Indian health programmes need to emphasise that attaining a healthy BMI before and during pregnancy in the context of ‘pre-conception health’ to prevent GDM is important, and this is consistent with previous evidence^(4,29,30)^. The HDU pattern-lower GDM association was consistent with national and global diet recommendations to increase diet diversity for optimal health^(31,32)^. The HDU and RFCS patterns consisting of both healthy and unhealthy foods may indicate low awareness about micronutrient-rich foods and the need to invest in public education. National surveys also show that a majority of Indians have suboptimal diets with excess cereals and not enough micronutrient-rich foods including fruits, vegetables, legumes, milk, poultry, meat and fish, which can be improved by public education^(31)^. Higher SES was positively associated with diet diversity (HDU and HTV), which highlights the need for national policies to make wholegrains, fruits, vegetables, dairy products and poultry foods more affordable^(4,19,32)^.

While the new Indian dietary guidelines define ‘a diverse diet as a balanced diet’^(31)^, it may not be practically achievable across all SES levels in a population of 1·3 billion. Achieving the suggested ‘balanced diet’ recommendations may cost a minimum of INR 15 000/month (GBP 148) for a family of four. This is possible for people of higher SES, challenging for those of middle SES and nearly impossible for those of lower SES (a majority). In this scenario, low-cost novel food-based evidence should be explored; the SARAS study showed that culturally appropriate, micronutrient-rich snacks (samosas with greens, fruit and milk) costing INR 5 (4 pence) per woman per d, given pre-conceptionally can prevent GDM^(33)^. This amounts to INR 2250 (GBP 22) per reproductive-aged woman for 450 d (6 months of pre-conception and 9 months of pregnancy) – feasible within national policy budgets^(34)^.

### Evidence before this study

Studies in high-income countries suggest that healthy diet patterns (higher intake of wholegrains, fruits and vegetables) are associated with a lower risk of GDM, and unhealthy diet patterns (higher intake of refined grains, fried and fast food, high sugar, red and processed meat) with a higher risk. Definitions of healthy/unhealthy diet patterns are heterogeneous across the globe. Evidence from low- and middle-income countries is sparse.

### Added value of this study

BANGLES is the first prospective study to investigate diet pattern–GDM associations in India. Three distinct peri-conceptional diet patterns were identified that were associated with socio-economic characteristics. BMI was strongly related to GDM risk and possibly mediated diet–GDM associations. The HDU pattern-lower GDM association was consistent with national and global recommendations to increase diet diversity.

### Implications of available evidence

First, most diet patterns were not clearly healthy or unhealthy; this may indicate low awareness among Indian women about healthy *v*. unhealthy foods. Hence, investing in public education about healthy foods is critical. Second, there cannot be a ‘universal healthy diet pattern’ for public health recommendations, in a large, diverse country like India. Future work could use pooled data to study an array of diet patterns across India. Third, global evidence suggests that ‘diet diversity’ is important for preventing non-communicable diseases. Higher SES in our study and others was associated with greater diet diversity, and national policies should aim to make micronutrient-rich foods including wholegrains, fruits, vegetables, dairy products, nuts and poultry more affordable.

## Supplementary material

For supplementary material accompanying this paper visit https://doi.org/10.1017/S1368980022001288. click here to view supplementary material
