# Supplementary material for: Peri-conceptional diet patterns and the risk of gestational diabetes mellitus in South Indian women
Source: Public Health Nutr. 2022 May 27;26(4):779–91. doi: 10.1017/S1368980022001288 (PMC10131144; doi:10.1017/S1368980022001288)

**SUPPLEMENTARY SECTIONS**

**INDEX OF SUPPLEMENTARY SECTIONS**

**SUPPLEMENTARY SECTION 1: The BANGLES FFQ………………………………………1**

**SUPPLEMENTARY SECTION 2: GLOSSARY OF INDIAN FOODS IN THE BANGLES FFQ…………………………………………………………………………………………………22**

**SUPPLEMENTARY SECTION 3: MODIFICATIONS USED FOR THE BANGLES FFQ AND THEIR RATIONALE………………………………………………………………….…...29**

**SUPPLEMENTARY SECTION 4: SUMMARY OF FOOD FREQUENCY QUESTIONNAIRE VALIDATION……………………………………….…………………….31**

**SUPPLEMENTARY SECTION 5: 55 FOOD-GROUPS, THEIR COMPONENTS AND MODIFICATIONS TO CONSTRUCT THE 68 FOOD-GROUPS ………………………….. 33**

**SUPPLEMENTARY SECTION 6: PCA OF ALL FOODS……………………………………40**

**SUPPLEMENTARY SECTION 7: Figure: Scree plot from the PCA of the 68 food-groups (n=785) ……………………………………………………………………………………………..46**

**SUPPLEMENTARY SECTION 8: Table 3: Medians and IQR for weekly frequencies of intake of key foods according to quintiles of diet patterns scores (n=785) …………………….46**

**SUPPLEMENTARY SECTION 9: Figure (d): Correlations of the first five diet patterns derived by PCA of 205 foods and 68 food-groups (n=785)……………………………………...49**

**SUPPLEMENTARY SECTION 1: THE BANGLES FFQ**

**Date of recording D M Y**

**Study Participant ID number:**

**Study Participant Name:  Interviewer code**

**TIME PERIOD:**

**The FFQ should be filled for the woman’s dietary pattern during the month before she knew she was pregnant. Ask the woman to think back to what she was eating during the month before she knew she was pregnant.**

**FREQUENCY**

**D1 = Once daily, D2 = Twice daily, D3 = 3 Times daily etc.**

**W1 = Once weekly, W2 = Twice weekly, W3 = 3 times weekly, W4 = 4 times weekly etc.**

**M1 = Once monthly, M2 = Twice monthly, M3 = 3 times monthly**

| **B1 = Small bowl, B2 = Medium bowl, B3 = Large bowl, B4 = Extra-large bowl** | **C = CIRCUMFERENCE (1- 3)**  **C1 = small**  **C2 = medium**  **C3 = large** |  |
| --- | --- | --- |
| **G1 = Small glass, G2 = Medium glass, G3 = Large glass** | **S = Small, M = Medium, L = Large** |  |
| **M2 = large mug** | **Pc1 = Chicken fry; Paneer Tikka**  **Pc2 = Fish Fry**  **Pc3 = Fish cutlet**  **= Lamb, Beef, Pork cutlet** |  |
| **PL1 = small plate,** | **Cu 1 = Cucumber 1**  **Cu 2 = Cucumber 2**  **Cu 3 = Cucumber 3** |  |
| **S1 = small sambhar spoon, S2 = medium sambhar spoon** |  |  |
| **TS2 = medium teaspoon, TS3 = large teaspoon, TS4 = extra-large teaspoon** | **THICKNESS:** |  |
| **O2 = medium ghee / oil spoon** | **T1 = THIN**  **T2 = THICK** |  |
| **NUMBER OF UNITS**  **Please enter whole numbers or decimals in this box (e.g. 0.5, 0.25). NOT fractions (e.g. 1/2,1/4)** | | |

**Circle the appropriate measure**

**DON’T WRITE IN GREY SHADED AREA**

|  | **ITEM** | **FREQUENCY** | **UNIT OF MEASURE** | **NO**  **OF UNITS** | **SIZE** | **THICK-NESS** |
| --- | --- | --- | --- | --- | --- | --- |
| 1. **BEVERAGES** | | | | | | |
| **1.** | **TEA** |  | **M2** |  |  |  |
| **2.** | **COFFEE** |  | **M2** |  |  |  |
| **3.** | **NORMAL COW’S MILK (AS PLAIN MILK / HORLICKS ETC.)** |  | **G3** |  |  |  |
| **4.** | **SKIMMED COW’S MILK (AS PLAIN MILK / HORLICKS ETC.)** |  | **G3** |  |  |  |
| **5.** | **BUTTERMILK** |  | **G3** |  |  |  |
| **6.** | **HORLICKS, COMPLAN, BOOST,**  **BOURNVITA** |  | **TS4** |  |  |  |
| **7.** | **PRO PL** |  | **TS4** |  |  |  |
| **8.** | **BABY AND ME** |  | **TS4** |  |  |  |
| **9.** | **PRISTINE BALANCE PL** |  | **TS4** |  |  |  |
| **10.** | **NUTRI RIGHT MOM** |  | **TS4** |  |  |  |
| **11.** | **GRD SMART** |  | **TS4** |  |  |  |
| **12.** | **GLUCON D POWDER** |  | **TS3** |  |  |  |
| **13.** | **HERSHEYS SYRUP / CHOCO SYRUP** |  | **TS3** |  |  |  |
| **14.** | **BADAM MILK** |  | **G3** |  |  |  |
| **15.** | **ELECTORAL** |  | **G3** |  |  |  |
| **16.** | **TENDER COCONUT WATER** |  | **G3** |  |  |  |
| **17.** | **SUGARCANE JUICE** |  | **G3** |  |  |  |
| **18.** | **PACKAGED FRUIT JUICE**  **(REAL, TROPICANA ETC.)** |  | **G3** |  |  |  |
| **19.** | **FRESH VEGETABLE SOUPS** |  | **B4** |  |  |  |
| **20.** | **FRESH NON – VEG SOUPS** |  | **B4** |  |  |  |
| **21.** | **READY TO MAKE SOUPS** |  | **B4** |  |  |  |
| **22.** | **COKE/ PEPSI / SPRITE / MIRINDA / FANTA OR OTHER FIZZY DRINK** |  | **G3** |  |  |  |
| **23.** | **FROOTI / MAAZA / SLICE / NIMBOOZ / APPY FIZZ** |  | **G3** |  |  |  |
| **24.** | **SQUASHES / SYRUPS (TANG/KISAN/ RASNA/ ROOHAFZA/LEMONADE)** |  | **G3** |  |  |  |
| **25.** | **FRESH FRUIT JUICE**  ***SPECIFY FRUIT:*** |  | **G3** |  |  |  |
| **26.** | **MILKSHAKES** |  | **G3** |  |  |  |
| **27.** | **OTHER BEVERAGE 1, **SPECIFY*:** |  | **G3** |  |  |  |
| **SECTION B: FRUIT** | | | | | | |
| **28.** | **APPLE** |  | **QUANTITY** |  | **S**  **M**  **L** |  |
| **29.** | **BANANA (PACCHE/**  **ROBUSTA/NENDRA KERALA TYPE)** |  | **QUANTITY** |  |  |  |
| **30.** | **BANANA (YELAKKI)** |  | **QUANTITY** |  |  |  |
| **31.** | **SWEET LIME** |  | **QUANTITY** |  |  |  |
| **32.** | **GUAVA** |  | **QUANTITY** |  | **S**  **M**  **L** |  |
| **33.** | **POMEGRANATE** |  | **B3** |  |  |  |
| **34.** | **SAPOTA** |  | **QUANTITY** |  |  |  |
| **35.** | **GRAPES (GREEN)** |  | **QUANTITY (NUMBER)** |  |  |  |
| **36.** | **MUSK MELON** |  | **B3** |  |  |  |
| **37.** | **PEAR** |  | **QUANTITY** |  | **S**  **M**  **L** |  |
| **38.** | **PAPAYA** |  | **B3** |  |  |  |
| **39.** | **PINEAPPLE** |  | **B3** |  |  |  |
| **40.** | **MIXED FRUIT SALAD** |  | **B3** |  |  |  |
| **41.** | **OTHER 1**  ***SPECIFY*: *** |  |  |  | **S**  **M**  **L** |  |
| **SECTION C: DRIED FRUIT / NUTS** | | | | | | |
| **42.** | **DATES** |  | **QUANTITY**  **(NUMBER)** |  |  |  |
| **43.** | **RAISINS** |  | **QUANTITY** |  |  |  |
| **44.** | **FIGS** |  | **QUANTITY** |  |  |  |
| **45.** | **PRUNES** |  | **QUANTITY** |  |  |  |
| **46.** | **CASHEW NUT** |  | **QUANTITY** |  |  |  |
| **47.** | **ALMOND** |  | **QUANTITY** |  |  |  |
| **48.** | **PISTACHIOS** |  | **QUANTITY** |  |  |  |
| **49.** | **APRICOTS** |  | **QUANTITY** |  |  |  |
| **50.** | **SUNFLOWER SEEDS** |  | **PS1** |  |  |  |
| **51.** | **PUMPKIN SEEDS** |  | **PS1** |  |  |  |
| **52.** | **FLAX SEEDS** |  | **PS1** |  |  |  |
| **53.** | **GROUNDNUTS** |  | **PS1** |  |  |  |
| **54.** | **OTHER 1, *SPECIFY*:**  ***probe for walnuts** |  |  |  |  |  |
| **SECTION D: RICE FOODS (Please read out all foods first before writing down frequency and amount: 55 - 62)** | | | | | | |
| **55.** | **PLAIN/BOILED RICE** |  | **B3** |  |  |  |
| **56.** | **RED RICE** |  | **B3** |  |  |  |
| **57.** | **BROWN RICE** |  | **B3** |  |  |  |
| **58.** | **RICE WITH DAL / SAMBHAR** |  | **B3** |  |  |  |
| **59.** | **RICE WITH GLV**  **SPECIFY GLV:** |  | **B3** |  |  |  |
| **60.** | **COLOUR / SEASONED RICE (TAMARIND / LEMON / GHEE / TOMATO)** |  | **B3** |  |  |  |
| **61.** | **VEGETABLE BATH / BISI BELE BATH / VEG KHICHDI** |  | **B3** |  |  |  |
| **62.** | **CURD RICE** |  | **B3** |  |  |  |
| **63.** | **RICE VERMICELLI / AVALAKKI/ PUFFED RICE (MANDAKKI)** |  | **B3** |  |  |  |
| **64.** | **IDLY** |  | **QUANTITY** |  | **S**  **L** |  |
| **65.** | **PLAIN DOSA** |  | **QUANTITY** |  | **C** | **T1**  **T2** |
| **66.** | **MASALA DOSA** |  | **QUANTITY** |  | **C** | **T1**  **T2** |
| **67.** | **RICE ROTI** |  | **QUANTITY** |  | **C** | **T1**  **T2** |
| **68.** | **RICE PUTTU/KADABU** |  | **B3** |  |  |  |
| **69.** | **OTHER 1, *SPECIFY*:** |  |  |  |  |  |
| **70.** | **CHAPATHI** |  | **QUANTITY** |  | **C** | **T1**  **T2** |
| **71.** | **POORI** |  | **QUANTITY** |  |  |  |
| **72.** | **PARANTHA** |  | **QUANTITY** |  | **C** | **T1**  **T2** |
| **73.** | **SWEET BUN / KARA BUN** |  | **QUANTITY** |  |  |  |
| **74.** | **SLICED WHITE BREAD** |  | **SLICE** |  |  |  |
| **75.** | **SLICED BROWN BREAD** |  | **SLICE** |  |  |  |
| **76.** | **OTHER BREADS**  ***SPECIFY*:** |  | **SLICE** |  |  |  |
| **77.** | **UPPUMA** |  | **B3** |  |  |  |
| **78.** | **BROKEN WHEAT / DALIA** |  | **B3** |  |  |  |
| **79.** | **WHEAT FLAKES** |  | **B4** |  |  |  |
| **80.** | **PASTA / MACARONI** |  | **B3** |  |  |  |
| **81.** | **SPAGETTI / NOODLES** |  | **B3** |  |  |  |
| **82.** | **OTHER 1, *SPECIFY*:** |  | **B3** |  |  |  |
| **SECTION E: RAGI FOODS** | | | | | | |
| **83.** | **RAGI ROTI** |  | **QUANTITY** |  | **C** | **T1**  **T2** |
| **84.** | **RAGI BALL** |  | **B2** |  |  |  |
| **85.** | **RAGI DOSA** |  | **QUANTITY** |  | **C** | **T1**  **T2** |
| **86.** | **RAGI MALT / MULTIGRAIN MALT / GANJI** |  | **G3** |  |  |  |
| **87.** | **RAGI VERMICELLI** |  | **B3** |  |  |  |
| **SECTION F: READY TO EAT BREAKFAST CEREALS** | | | | | | |
| **88.** | **OAT + MILK** |  | **B4** |  |  |  |
| **89.** | **OATS + WATER** |  | **B4** |  |  |  |
| **90.** | **MUESLI** |  | **B4** |  |  |  |
| **91.** | **ALL BRAN FLAKES** |  | **B4** |  |  |  |
| **92.** | **CORN FLAKES (PLAIN)** |  | **B4** |  |  |  |
| **93.** | **CORN FLAKES (WITH DRYFRUIT)** |  | **B4** |  |  |  |
| **94.** | **CHIA SEEDS** |  | **PS1** |  |  |  |
| **95.** | **OTHER 1, *SPECIFY*:** |  |  |  |  |  |
| **96.** | **URAD VADA** |  | **QUANTITY** |  |  |  |
| **97.** | **CHILAS / ADAI / PESARATTU** |  | **QUANTITY** |  |  |  |
| **SECTION G: SIDE PREPARATIONS** | | | | | | |
| **98.** | **LEGUME BASED DHAL**  **(GREEN GRAM / BENGAL GRAM/ CHANNA/ RAJMA)** |  | **PS2** |  |  |  |
| **99.** | **SPLIT DAL BASED DHAL**  **(MOONG/ TOOR/ MASOOR)** |  | **PS2** |  |  |  |
| **100.** | **POTATO PALYA** |  | **PS2** |  |  |  |
| **101.** | **CARROT PALYA** |  | **PS2** |  |  |  |
| **102.** | **MIX VEG SAMBHAR** |  | **S2** |  |  |  |
| **103.** | **GLV / SOPPU SAMBHAR** |  | **S2** |  |  |  |
| **104.** | **BEETROOT, RADISH, YAM, TAPIOCA PALYA** |  | **PS2** |  |  |  |
| **105.** | **LADYSFINGER PALYA** |  | **PS2** |  |  |  |
| **106.** | **CABBAGE / KNOL KHOL PALYA** |  | **PS2** |  |  |  |
| **107.** | **CAULIFLOWER PALYA** |  | **PS2** |  |  |  |
| **108.** | **CHOW CHOW, PUMPKIN, GOURDS** |  | **PS2** |  |  |  |
| **109.** | **CAPSICUM PALYA** |  | **PS2** |  |  |  |
| **110.** | **DRUMSTICK PALYA** |  | **PIECES** |  |  |  |
| **111.** | **BRINJAL PALYA** |  | **PS2** |  |  |  |
| **112.** | **BEANS PLAYA** |  | **PS2** |  |  |  |
| **113.** | **MUSHROOM PALYA** |  | **PS2** |  |  |  |
| **114.** | **GLV / SOPPU PALYA**  **SPECIFY GLV CODE:** |  | **PS2** |  |  |  |
| **115.** | **MULTI VEGETABLE SAAGU** |  | **S2** |  |  |  |
| **116.** | **RASAM** |  | **S2** |  |  |  |
| **117.** | **OTHER 1, *SPECIFY*** |  |  |  |  |  |
| **SECTION H: RAW VEGETABLES** | | | | | | |
| **118.** | **CUCUMBER**  **(without skin)** |  |  |  | **Cu 1**  **Cu 2**  **Cu 3** |  |
| **119.** | **TOMATO** |  | **QUANTITY** |  |  |  |
| **120.** | **ONION** |  | **QUANTITY** |  |  |  |
| **121.** | **CARROT** |  | **QUANTITY** |  |  |  |
| **122.** | **RADISH** |  | **QUANTITY** |  |  |  |
| **123.** | **KOSAMBARI** |  | **B1** |  |  |  |
| **124.** | **RUNNER BEANS** |  | **QUANTITY** |  |  |  |
| **125.** | **OTHER 1, *SPECIFY*:** |  |  |  |  |  |
| **SECTION I: PREPARATION (NON VEGETARIAN)** | | | | | | |
| **126.** | **CHICKEN BIRIYANI** |  | **B3** |  |  |  |
| **127.** | **CHICKEN FRY (ROASTED/GRILLED)** |  | **Pc1** |  |  |  |
| **128.** | **CHICKEN CURRY** |  | **B2** |  |  |  |
| **129.** | **FISH FRY** |  | **Pc2** |  |  |  |
| **130.** | **FISH CURRY** |  | **S2** |  |  |  |
| **131.** | **FISH CUTLET** |  | **Pc3** |  |  |  |
| **132.** | **DRIED FISH / DRIED SEAFOOD** |  | **PS2** |  |  |  |
| **133.** | **PRAWN, CRAB, SHELLFISH** |  | **PS2** |  |  |  |
| **134.** | **LAMB, BEEF, PORK- FRY** |  | **PS2** |  |  |  |
| **135.** | **LAMB, BEEF, PORK CUTLET** |  | **Pc3** |  |  |  |
| **136.** | **LAMB, BEEF, PORK CURRY** |  | **B2** |  |  |  |
| **137.** | **LIVER, BRAIN, KIDNEY** |  | **PS2** |  |  |  |
| **138.** | **KEEMA CURRY** |  | **PS2** |  |  |  |
| **139.** | **HAM / SALAMI / BACON** |  | **SLICE** |  |  |  |
| **140.** | **MUTTON BIRIYANI** |  | **B3** |  |  |  |
| **141.** | **EGG BIRIYANI** |  | **B3** |  |  |  |
| **142.** | **EGG - BOILED**  **(WITH YOLK)** |  | **QUANTITY** |  |  |  |
| **143.** | **EGG - BOILED**  **(WITHOUT YOLK)** |  | **QUANTITY** |  |  |  |
| **144.** | **EGG BURCHI, CURRY** |  | **PS2** |  |  |  |
| **145.** | **OMELETTE WITH YOLK** |  | **QUANTITY** |  |  |  |
| **146.** | **OMELETTE WITHOUT YOLK** |  | **QUANTITY** |  |  |  |
| **147.** | **OTHER 1, **SPECIFY*:** |  |  |  |  |  |
| **148.** | **OTHER 2, **SPECIFY*:** |  |  |  |  |  |
| **SECTION J: JAM / CHUTNEY** | | | | | | |
| **149.** | **PICKLE (LEMON / MANGO)** |  | **TS3** |  |  |  |
| **150.** | **COCONUT CHUTNEY** |  | **PS1** |  |  |  |
| **151.** | **GROUNDNUT CHUTNEY** |  | **PS1** |  |  |  |
| **152.** | **KETCHUP** |  | **TS3** |  |  |  |
| **153.** | **JAM** |  | **TS3** |  |  |  |
| **154.** | **HONEY** |  | **TS3** |  |  |  |
| **155.** | **OTHER 1, **SPECIFY*:** |  | **TS3** |  |  |  |
| **SECTION K: ADDED SUGAR** | | | | | | |
| **156.** | **SUGAR IN FRUIT JUICE** |  | **TS2** |  |  |  |
| **157.** | **SUGAR IN TEA** |  | **TS2** |  |  |  |
| **158.** | **SUGAR IN COFFEE** |  | **TS2** |  |  |  |
| **159.** | **SUGAR IN MILK** |  | **TS2** |  |  |  |

|  | **ITEM** | **FREQUENCY** | **UNIT OF MEASURE** | **NO**  **OF UNITS** | **SIZE** | **THICK-**  **NESS** |
| --- | --- | --- | --- | --- | --- | --- |
| **SECTION L: SAVOURY (SALTED) SNACKS** | | | | | | |
| **161.** | **PAPAD/ SANDIGE (HOME FRIED)** |  | **QUANTITY** |  |  |  |
| **162.** | **NAMKEEN/MIXTURES/ SAVOURIES** |  | **B3** |  |  |  |
| **163.** | **CHAKLI/MURUKKU/**  **KODBALE** |  | **QUANTITY** |  |  |  |
| **164.** | **PAKODA/ BONDA/**  **BHAJJI/ SAMOSA** |  | **QUANTITY** |  |  |  |
| **165.** | **BABYCORN / GOBI MANCHURIAN** |  | **P1** |  |  |  |
| **166.** | **BOILED / ROASTED CORN** |  | **B3** |  |  |  |
| **167.** | **CHIPS (POTATO/PLANTAIN)**  **FRENCH FRIES** |  | **B3** |  |  |  |
| **168.** | **PUFF** |  | **QUANTITY** |  |  |  |
| **169.** | **OTHER 1, *SPECIFY*:** |  |  |  |  |  |
| **SECTION M: SWEET SNACKS** | | | | | | |
| **170.** | **CHOCOLATE**  ***SPECIFY BRAND:** |  | **QUANTITY** |  | **S**  **M**  **L** |  |
| **171.** | **TOFFEE/ CANDY** |  | **NUMBER** |  |  |  |
| **172.** | **CAKE WITHOUT CREAM** |  | **QUANTITY** |  |  |  |
| **173.** | **CAKE WITH CREAM / PASTRY** |  | **QUANTITY** |  |  |  |
| **174.** | **SWEET BISCUITS**  **(EG: PARLE G / GLUCOSE / TIGER / MARIE / GOODDAY)** |  | **QUANTITY** |  |  |  |
| **175.** | **SALTED BISCUITS**  **EG: MONACO / 50 – 50 / KRACKJACK / MASKA CHASKA** |  | **QUANTITY** |  |  |  |
| **176.** | **CREAM BISCUITS**  **EG: DREAM CREAM, MILKY BITE, JIM JAM, BOURBON, BRITANNIA PURE MAGIC** |  | **QUANTITY** |  |  |  |
| **177.** | **KESARIBAATH / RAVA KESARI/ SHEERA/ SUJI HALWA** |  | **B2** |  |  |  |
| **178.** | **OBBATTU/ HOLIGE / BOLI/ BOBATLU/ PURANPOLI** |  | **QUANTITY** |  |  |  |
| **179.** | **BARFI /PEDHA** |  | **QUANTITY** |  |  |  |
| **180.** | **GULAB JAMOON, JALEBI. JANGIR** |  | **QUANTITY** |  |  |  |
| **181.** | **CHIKKI** |  | **QUANTITY** |  |  |  |
| **182.** | **LADDOO, MYSORE PAK** |  | **QUANTITY** |  |  |  |
| **183.** | **HALWA (DUMROOT/CARROT)** |  | **QUANTITY** |  |  |  |
| **184.** | **KHEER / PAYASAM** |  | **B2** |  |  |  |
| **185.** | **BENGALI SWEETS (RASGULLA / RASMALAI / CHUM CHUM / CHAMPAKALI/ MALAI**  **SANDWICH)** |  | **QUANTITY** |  |  |  |
| **186.** | **PUDDING/CUSTARD/SOUFFLE/CHEESE CAKE** |  | **B2** |  |  |  |
| **187.** | **OTHER SWEETS OR SNACKS, **SPECIFY:*** |  |  |  |  |  |
| **SECTION N: FAST FOOD** | | | | | | |
| **188.** | **CHATS**  **MASALA PURI, PANI PURI, BHEL PURI** |  |  | **1** |  |  |
| **189.** | **VEG PIZZA** |  | **SLICES** |  | **6’’ (PERSONAL)**  **12’’ (MEDIUM)** |  |
| **190.** | **NON VEG PIZZA** |  | **SLICES** |  | **6’’ (PERSONAL)**  **12’’ (MEDIUM)** |  |
| **191.** | **VEG BURGER** |  | **QUANTITY** |  | **SMALL**  **LARGE** |  |
| **192.** | **NON VEG BURGER** |  | **QUANTITY** |  | **SMALL**  **LARGE** |  |
| **193.** | **OTHER FAST FOOD, *SPECIFY*:** |  |  |  |  |  |
| **SECTION O: MILK PRODUCTS** | | | | | | |
| **194.** | **CURD** |  | **S1** |  |  |  |
| **195.** | **RAITHA (ANY VEGETABLE RAITA)** |  | **S1** |  |  |  |
| **196.** | **BUTTER** |  | **TS3** |  |  |  |
| **197.** | **GHEE ON THE PLATE/ TABLE** |  | **O2** |  |  |  |
| **198.** | **CHEESE AS: (GRATED CHEDDAR/MOZARELLA, SPREAD AND ALL TYPES)** |  | **PS1** |  |  |  |
| **199.** | **PANEER CURRY**  **(PANEER BUTTER MASALA/**  **KADAI PANEER)** |  | **B2** |  |  |  |
| **200.** | **PANEER TIKKA** |  | **Pc1** |  |  |  |
| **201.** | **ICECREAMS**  ***include cup & stick icecreams** |  |  | **X 1** |  |  |
| **202.** | **OTHERS SPECIFY: *** |  |  |  |  |  |

**SECTION P:**

**203. HOW MUCH OIL IS USED / PURCHASED FOR FOOD PREPARATION AND COOKING (PER MONTH? ________________________LITRES.**

**204. WHICH BRAND OF OIL DO YOU USE? _____________________________________**

**205. HOW MUCH GHEE IS PURCHASED FOR COOKING? ____________________ Kg.**

|  |
| --- |

**206. HOW MANY COCONUTS ARE USED PER MONTH?**

**207. HOW MUCH DALDA DO YOU USE PER MONTH? ___________________ Kg.**

**208. WHICH BRAND OF SALT DO YOU BUY/ USE FOR COOKING? ________________**

**209. HOW MANY SALT PACKETS (1 Kg) DO YOU PURCHASE PER MONTH? ________**

**210. HOW MANY PERSONS ARE THERE IN THE FAMILY? ______________________**

**SECTION Q (SEASONAL FOODS)**

**Do you consume any of these foods during the season?**

|  | **ITEM** | **FREQUENCY** | **UNIT OF MEASURE** | **NO**  **OF UNITS** | **SIZE** | **THICK-**  **NESS** |
| --- | --- | --- | --- | --- | --- | --- |
| **211.** | **RAW MANGO** |  | **QUANTITY** |  | **S**  **M**  **L** |  |
| **212.** | **RIPE MANGO** |  | **QUANTITY** |  |  |  |
| **213.** | **JACKFRUIT** |  | **SEGMENT** |  |  |  |
| **214.** | **AVRE KALU (PALYA / SAMBHAR / SNACKS / UPMA)** |  | **B3** |  |  |  |
| **215.** | **ORANGE** |  | **QUANTITY** |  |  |  |
| **216.** | **GREEN PEAS (PALYA / SAMBHAR / SNACKS / UPMA)** |  | **B3** |  |  |  |
| **217.** | **GRAPES (PURPLE)** |  | **QUANTITY** |  |  |  |
| **218.** | **WATERMELON** |  | **B3** |  |  |  |
| **219.** | **LITCHI** |  | **QUANTITY** |  |  |  |
| **220.** | **AMLA** |  | **QUANTITY** |  |  |  |
| **221.** | **JAMUN** |  | **QUANTITY** |  |  |  |
| **222.** | **PLUM** |  | **QUANTITY** |  |  |  |
| **223.** | **CUSTARD APPLE** |  | **QUANTITY** |  |  |  |
| **224.** | **STRAWBERRY** |  | **QUANTITY** |  |  |  |

**OTHERS * (PROBE LIST)**

**BEVERAGES:** SOY MILK (G3)**,** COCONUT MILK (G3)

**FRUITS:** KIWI (QUANTITY), AVOCADO (QUANTITY)**,** ICE APPLE (QUANTITY)

**SWEETS:** BADUSHA (QUANTITY)**,** KAJJAYA (QUANTITY)**,** DOUGHNUTS (QUANTITY)

**VEGETABLES:** LETTUCE (B3)**,** BROCCCOLI (B3)

**JAMS & JELLIES:** PEANUT BUTTER(TS3)**,** MAYONNAISE (TS3)

**SUPPLEMENTARY SECTION 2:** **GLOSSARY OF INDIAN FOODS IN THE BANGLES FFQ (parts adapted and modified from Indian food composition tables ^a^**

| **Buttermilk** | A popular south Indian savoury drink prepared from whipped curd diluted with water and salt. Asafoetida and curry leaves are optional. |
| --- | --- |
| **Horlicks, complan, boost, bournvita, Pro PL, baby & me, Pristine Balance PL, Protovic DF, Nutriright Mom, GRD Smart** | Types of nutrient enriched, sweetened supplement powders consumed with mostly milk or at times hot water. |
| **Hersheys syrup / choco syrup** | Concentrated sweetened chocolate flavoured syrup for milk and used with milk and on desserts. |
| **Badam milk** | Traditional milk drink prepared by boiling milk with ground almonds, sugar, saffron and cardamom. |
| **Sugarcane juice** | Sugarcane juice is the liquid extracted from pressed [sugarcane](https://en.wikipedia.org/wiki/Sugarcane). |
| **Squashes / syrups (tang/kisan/ rasna/ roohafza/lemonade)** | Concentrated flavoured sweetened syrups made by diluting water. |
| **Red rice** | Kerala Matta rice is an indigenous variety of rice grown in [Palakkad](https://en.wikipedia.org/wiki/Palakkad) district of [Kerala](https://en.wikipedia.org/wiki/Kerala), [India](https://en.wikipedia.org/wiki/India). It is different from [brown rice](https://en.wikipedia.org/wiki/Brown_rice). It is popular in Kerala and coastal Karnataka in India. |
| **Brown rice** | Unpolished variety of rice with outer layer intact. |
| **Rice with dal / sambhar** | A staple dish in Karnataka where rice mixed with sambhar (cooked with red gram dal, vegetables, tamarind pulp and spices). |
| **Rice with glv** | Also known as green pulao or green leafy vegetable is prepared with rice cooked with green leafy vegetables and spices. |
| **Spice Seasoned/ flavoured rice (tamarind / lemon / ghee / tomato)** | Spice seasoned rice are varieties of rice consumed for breakfast or lunch usually. It is prepared by seasoning cooked rice with oil, spices and tamarind pulp/lemon juice/tomatoes. |
| **Vegetable bath or pulao / bisi bele bath / veg khichdi** | Pulao or vegetable baath is prepared with buttered long grain or aromatic basmati rice cooked with vegetables, spices, nuts and flavours. Bisibelebaath is a popular main course dish in the southern state of Karnataka. It is prepared by cooking rice, red gram dal, vegetables and tamarind pulp with ground spicy masala including dry coconut/ roasted coconut. Served hot with ghee. Khichdi is prepared by pressure cooking rice with green gram dhal, red gram dhal, vegetables, spices and ghee. |
| **Curd rice** | A traditional rice dish made by mixing curds, cooked rice, onion and spices. |
| **Rice vermicelli / avalakki/ puffed rice (mandakki)** | Rice vermicelli also known as semiya is similar to Idiyappam. It is prepared by sautéing with onion, spices and lemon juice and sometimes coconut or other vegetables.  Puffed rice is eaten as a breakfast or evening snack. It is softened with water and cooked with oil, onion, spices and this dish is called ‘mandakki’ in Karnataka. |
| **Idly** | A traditional breakfast popular staple food in southern Indian states, in recent times prepared from ready batters in urban areas. Idly is a spongy steamed rice cake made from a fermented batter of rice and black gram dal in a desired proportion. |
| **Plain dosa** | Dosa is made from fermented batter of parboiled rice and split black gram and cooekd in shallow pan called tava with oil or without oil in non-stick pan. There is a filling with a spicy potato stir-fry inside the masala dosa. |
| **Masala dosa** |  |
| **Rice roti** | A traditional bread rice [roti](http://www.indianfoodsite.com/breads.htm#ROTI) is made from rice flour, warm water, spices and sometimes greens. |
| **Rice puttu/ kadabu** | Puttu is a popular breakfast dish in Kerala and Tamil Nadu. It is a steamed rice flourcake prepared using a specially designed puttu mould and served with grated coconut, sugar or without sugar and ripe banana at times. |
| **Chapati** | Dough made of wheat flour, salt, water rested for 30 minutes and then rolled into flat breads and cooked on both sides with oil/ ghee in tava. |
| **Poori** | Wheat flour (refined or whole wheat) kneaded hard or tightly is rolled into small circlets and deep fried in oil. |
| **Parantha** | A popular traditional main course in Punjab state popular all over India. Soft dough prepared from whole wheat flour is rolled into chapathi and stuffed with cooked vegetables such as methi leaves, cauliflower, potato, onion and shallow fried on gridle pan with oil/ ghee/ butter. |
| **Sweet bun / kara bun** | Kara bun is a type of bun made with spices such as fennel, chillies, onion etc. |
| **Uppuma** | Uppuma is a traditional south Indian breakfast item, prepared by stir frying followed by boiling semolina/ sago in water with the addition of onion, salt and spices. |
| **Broken wheat / dalia** | Broken wheat is made by milling whole raw wheat grains coarsely. It is boiled with water like rice and eaten with lentil and vegetable side dishes. |
| **Spagetti / noodles** | A Chinese cuisine staple, popular in several parts of India. It is prepared by stir frying the noodles with vegetables such as carrot, capsicum, cabbage with sauce and spices. |
| **Ragi roti** | A traditional bread made from ragi, onion and spices or other millets such as bajra, jowar etc. |
| **Ragi ball** | A traditional semi-solid gruel prepared from finger millet or ragi are commonly consumed as breakfast choices especially in Karnataka known localy as ‘ragi mudde’ in urban and mainly in rural areas. Ragi is boiled in water with salt to desired consistency and made into balls. |
| **Ragi dosa** | A type of pancake made with ragi flour, buttermilk and spices on a shallow pan. |
| **Ragi Malt / Multigrain Malt / Ganji** | Porridge of a thin consistency consumed for breakfast especially in rural areas. Ragi flour is boiled with water, milk (optional) and salt/sugar. In some parts of India buttermilk is also added. |
| **Ragi vermicelli** | A snack or breakfast dish made with vermicelli made out of ragi that is boiled and cooked with onion and spices. |
| **Urad vada** | A popular snack in most parts of India, It is prepared with coarsely/ finely ground paste of split black gram and mixed with onion, coriander leaves, ginger, black pepper grits, asafoetida and hand pressed into circular shape with a hole in the centre for even cooking and then deep fried in oil. Vadas are also made sometimes with Bengal gram dal, onions, spices and deep fried. |
| **Chilas / adai / pesarattu** | Traditional breakfast/ dinner main dish from the state of Tamil Nadu. It is a thick type of dosa (pancake) made out of coarsely ground batter of rice and pulses and is cooked on a hot tava/ griddle with oil. |
| **Legume based dhal** | A side dish prepared by boiling whole legumes like Bengal gram, rajmah, cowpeas, chickpeas with salt and seasoned with spices and onion. |
| **Split dal based dhal** | A side dish prepared by boiling split dhal from whole legumes like bengal gram dhal, moong dhal, red gram dhal etc. with salt and seasoned with spices and onion. |
| **Potato palya** | Palya is a term for vegetable side dishes in Kannada where vegetables are stir fried with spices and grated coconut. |
| **Carrot palya** |  |
| **Mixed veg sambhar** | Sambhar is a traditional side dish eaten often with rice and sometimes with idly. Made out of red gram dhal, tomatoes, onion, vegetables or green leafy vegatables alone - cooked din tamarind pulp along with spices. |
| **Glv / soppu sambhar** |  |
| **Multi vegetable saagu** | Saagu is a meal accompaniment with rice, chapathi, parantha etc. Many vegetables such as carrot, green peas, potato etc. are cooked with spices and coconut paste/ coconut milk to a thick consistency. |
| **Rasam** | In South Indian households rasam commonly refers to soup prepared with sweet-sour stock made from tamarind, along with tomato and lentil, added spices and garnish. |
| **Kosambari** | Kosambari is a typical south Indian [salad](https://en.wikipedia.org/wiki/Salad) made from [pulses](https://en.wikipedia.org/wiki/Pulse_(legume)) (split [legumes](https://en.wikipedia.org/wiki/Legumes)) and seasoned with [mustard seeds](https://en.wikipedia.org/wiki/Mustard_seed). |
| **Chicken biriyani** | A special occasion rice dish made with edible portions of chicken cooked with ground spices for flavouring and tossed with stir fried aromatic basmati rice. |
| **Chicken fry** | Edible portions of chicken cooked with spices and shallow fried or deep fried in oil or pan or kadai. |
| **Chicken curry** | Edible portions of chicken sautéed with ginger, garlic paste, spices and coconut or without coconut cooked in water to a thick consistency or gravy. |
| **Fish fry** | Edible portions of fish cooked with spices and shallow fried or deep fried in oil or pan or kadai. |
| **Fish curry** | Edible portions of fish sautéed with ginger, garlic paste, spices and coconut or without coconut cooked in water to a thick consistency or gravy or steamed and then fried. |
| **Lamb, beef, pork- fry** | Edible portions of mutton cooked with spices and shallow fried or deep fried in oil or pan or kadai. |
| **Lamb, beef, pork - cutlet** | Cutlet is a deep fried or shallow fried snack prepared by cooking meat by itself or with vegetables, spices, made into balls, flattened to desired shapes dipped into egg mix and coated with bread crumbs sometimes. |
| **Lamb, beef, pork - curry** | Edible portions of mutton sautéed with ginger, garlic paste, spices and coconut or without coconut cooked in water to a thick consistency or gravy or steamed and then fried. |
| **Keema curry** | A traditional dish of the Indian subcontinent, typically minced-lamb curry with peas or potatoes and spices. |
| **Mutton biriyani** | A special occasion rice dish made with edible portions of mutton cooked with ground spices for flavouring and tossed with stir fried aromatic basmati rice. |
| **Egg biriyani** | A special occasion rice dish made with edible portions of egg cooked with ground spices for flavouring and tossed with stir fried aromatic basmati rice. |
| **Egg burchi, curry** | It is a side dish eaten with chapathi, rice or western style breads. Egg burchi is made with egg scrambled in oil, onion, chillies and spices or a curry is made with spices and onion paste with boiled eggs cooked in the gravy. |
| **Pickle (lemon / mango)** | A preserved food popular as accompaniments in India. It is made of mango / lemon and garlic, tomato, chillies, mixed vegetables, prawn, fish and cooked with spices, slat and oil. |
| **Coconut chutney** | Chutney is a very common condiment used with Indian foods made of coconut, groundnut, mango, tomato or mint or any other vegetable, cooked and ground with salt and spices. |
| **Groundnut chutney** |  |
| **Other Chutney and Powders** | Chutney powders are preserved chutney powders made from dry ingredients such as groundnut, lentils and spices. |
| **Jaggery** | Jaggery is a [traditional](https://en.wikipedia.org/wiki/List_of_unrefined_sweeteners) [non-centrifugal cane sugar](https://en.wikipedia.org/wiki/Non-centrifugal_cane_sugar) consumed in some countries in [Asia](https://en.wikipedia.org/wiki/Asia) and the Americas. It is a concentrated product of [cane juice](https://en.wikipedia.org/wiki/Cane_juice) and often [date](https://en.wikipedia.org/wiki/Date_(fruit)) or [palm](https://en.wikipedia.org/wiki/Arecaceae) [sap](https://en.wikipedia.org/wiki/Plant_sap) (see: [palm sugar](https://en.wikipedia.org/wiki/Palm_sugar)) without separation of the [molasses](https://en.wikipedia.org/wiki/Molasses) and crystals, and can vary from golden brown to dark brown in colour. It contains up to 50% [sucrose](https://en.wikipedia.org/wiki/Sucrose), up to 20% [invert sugars](https://en.wikipedia.org/wiki/Invert_sugar), and up to 20% moisture, with the remainder made up of other insoluble matter, such as [wood ash](https://en.wikipedia.org/wiki/Wood_ash), [proteins](https://en.wikipedia.org/wiki/Protein), and [bagasse](https://en.wikipedia.org/wiki/Bagasse) fibres |
| **Papad/ sandige (home fried)** | Thin crispy Indian accompaniment made out of a dhal dough usually black gram dhal/ green gram dhal, rice and cumin seeds, rolled into a thin flatbread, shade sun dried and either roasted or deep fried in oil. |
| **Namkeen/mixtures/savouries** | It is a Bengal gram dhal flour based deep fried snack/ savoury/ namkeen commonly eaten in India. |
| **Chakli/murukku/kodbale** | Savoury snacks made from soft dough prepared with rice flour, split Bengal ram flour, cumin seeds, salt, butter and water. The dough is places in a mould, pressed and deep fried in oil into circular extruded shapes. |
| **Pakoda/bonda/bhajji/samosa** | Bonda / Pakoda/ bhajji are spicy fried snack popular in the southern states of India. A thick batter of besan (Bengal gram dal) is mixed with onion and green chillies, made into balls and deep fried in oil.  Samosa is a popular snack in India, especially in North. Dough made from refined wheat flour is folded into triangular shape wrap and stuffed with cooked mixture of potato, peas, carrot, onion and spices and deep fried in oil. |
| **Babycorn / gobi manchurian** | Gobi (cauliflower) manchurian is the result of the adaptation of Chinese cooking and seasoning techniques to suit Indian tastes. It has cauliflower fritters in comparatively dry sauce like texture and is typically served and enjoyed as a snack or starter with tomato ketchup as dipping sauce. |
| **Boiled / roasted corn** | Boiled sweetcorn topped with butter, salt or other spice flavours. |
| **Puff** | Puff is eaten as a snack. The primary ingredient is refined wheat flour and hydrogenated fat (vanaspathi) which are blended into a soft dough and folded into layers and further rolled and baked with cooked vegetables/ meat/ egg / chicken. |
| **Kesaribaath** | Kesaribaath is a sweet made from roasted wheat grits and cooked with sugar, water and flavoured with ghee, cardamom or saffron and cashew nuts. |
| **Obbattu/holige/boli/bobatlu/puranpoli** | A popular dish in Maharashtra, Gujrat, Karnataka, Andhra Pradesh and Tamil Nadu. Soft dough prepared with refined wheat flour rolled into a chapathi and stuffed with a mixture of Bengal ram dhal, sugar/ jiggery, nutmeg, cardamom, coconut and shallow fried with ghee. |
| **Barfi /pedha** | Pedha is a milk based sweet where milk is reduced with sugar and sugar and cooked at a simmering temperature till it separates from the sides of the pan. Addition of ghee and other flavourings like saffron, cardamom and flavours are optional.  Barfi is an Indian sweet made with ground coconut paste/ split Bengal gram flour, cooked with milk and sugar (or jaggery) and cooked till it separates from the sides of the pan, then spread on plate and cut into desired shapes. |
| **Gulab jamoon, jalebi. jangir** | Gulab jamoon: Milk based popular Indian sweet prepared with milk khoa and refined wheat flour to form a soft dough and made into small balls. It is then deep fried in oil or ghee and dipped in a sugar syrup.  Jalebi: It is made with black gram dhal batter squeezed through cone (similar to icing cone) nozzle into circular shapes and fried in oil. Dipped in sugar syrup and served. |
| **Chikki** | Chikki is a peanut/ groundnut based sweet popularly called chikki. It is made with groundnut, seasame seeds or any crushed nuts cooked in jaggery syprup rolled into balls or flat squares. |
| **Laddoo, mysore pak** | Mysore pak is Bengal gram flour ghee roasted and blended with hot sugar syrup and ghee and cooked till it leaves the sides of the pan. The mixture is poured onto a greased plate and cooled. Later sliced inti rectangular pieces.  Ladoo is a traditional sweet where batter of Bengal gram dhal flour is deep fried in oil using perforated ladle and this cooked boondhi is further mixed with sugar syrup, ghee, roasted cahew nuts and cardamom. This mixture is hand pressed into balls of desired sizes. |
| **Halwa (dumroot/carrot)** | A popular Indian sweet made from wheat flour, grated carrot, bottle gourd or semolina, cooked with sugar, ghee and water/ milk to a thick consistency. |
| **Kheer/Payasam** | Roasted vermicelli/ semolina/ green gram dhal/ carrot is cooked with milk and sugar/ jaggery and flavoured with ghee, cardamom and cashew nuts. |
| **Bengali sweets** | Bengali sweets are all made of reducing full cream milk into chenna or cottage cheese and mixing with saffron, sugar in different combinations. They are popular in all parts of India. |
| **Chats: pani puri, bhel puri etc.** | It is a north Indian savoury snack but popular all over India, especially in urban areas. Bhel poori is a mixture of puffed rice, sev (fried thins made of besan flour), salad vegetables (tomato, potato, onion and cucumber) and crisp pooris tossed with a tangy sweet and sour chutney and green chutney.  Pani puri is a hollow crisp puri made of semolina, refined flour, filled with tamarind juice, spice powders made of raw mango, cumin, coriander, black pepper, rock salt and chilli powder along with boiled mashed potato and chickpeas or green gram. |
| **Raitha (any vegetable raita)** | Raita is an accompaniment for chapathi/roti/rice based dishes in North India. Curd mixed with salad vegetables like onion, cucumber, carrot, tomato and seasoned with spices. |
| **Ghee on the plate/ table** | Ghee is clarified butter made by melting butter and separating the fat from the solids. |
| **Paneer curry(paneer butter masala/kadai paneer)** | An accompaniment for roti/ naan/ plain rice in North India. It is prepared with boiled and mashed green leafy vegetables or onion and tomato and cooked with paneer (Indian cottage cheese, garlic, ginger and other spices to a thick consistency. |
| **Paneer tikka** | Paneer tikka is an Indian dish made from chunks of paneer marinated in spices and grilled in a tandoor or shallow pan. |
| **Reference:**   1. SUDHA, V., MOHAN, V., ANJANA, R.M. & KRISHNASWAMY, K. 2016. *Dr Mohan’s Atlas of Indian Foods*. 1^st^ Edition. Chennai, India: Madras Diabetes Research Foundation publishers. | |

**SUPPLEMENTARY SECTION 3: MODIFICATIONS USED FOR THE BANGLES FFQ AND THEIR RATIONALE**

The pilot 24-hour recalls were used to finalise the BANGLES FFQ, making it culturally appropriate to pregnant women in Bangalore. The following changes were made from the Parthenon FFQ:

**Beverages:** Nutrition supplement powders containing energy, protein and micronutrients were frequently consumed among pregnant women with milk and were added to the FFQ. The most frequently consumed brands, which vary in their nutritional composition were added.

Powders such as Glucon D (Glucose) and Electroral (containing salt, sugar and minerals) were frequently consumed in the 1st trimester, by adding to water or tender coconut water. Hershey’s chocolate syrup was frequently consumed with milk and eaten with biscuits. In addition, sugarcane juice, packaged fruit juices, milkshakes, fresh vegetable, non-vegetarian and instant soups were added.

**Seasonal fruits:** A market survey in 6 shops in different areas of Bangalore indicated that, some fruits (guava, pomegranate, sapota and green grapes) were available throughout the year. Others (raw mango, ripe mango, jackfruit, orange, watermelon, purple grapes, litchi, amla, jamun, plum, custard apple and strawberry) were seasonal (for example, mango was available from March to June). Therefore, I created a separate FFQ section for seasonal foods. Rarely consumed foods like kiwi, avocado and ice apples were added to a probing list of ‘other’ foods. Seasonal foods were grouped separately in the FFQ and the women were asked how often they consumed them when in season, making it clear they were not consumed all-round the year. Hence, no correction factor was necessary during analysis.

**Dried fruits and nuts:** Piloting revealed that dried fruits, nuts and seeds are consumed by the current generation of reproductive-aged women that were not consumed a decade ago, and can be termed ‘new age foods’. These are marketed as ‘super-foods’ by the food industry. Figs, prunes, pistachios, apricots, sunflower seeds, pumpkin seeds, flax seeds, groundnuts and walnuts were added to the FFQ.

**Rice and wheat foods:** The rice and wheat foods sections were combined because they are consumed daily and often together (for example, chapathi and rice). By combining these sections, the nutritionists were able to probe and record the frequency more easily.

**Rice foods:** Seasoned rice varieties such as tamarind, onion, ghee and lemon rice were coupled in a single group because they are similar in nutritive value, with few calories coming from the seasoning ingredients, and the majority of calories coming from the rice itself. Red and brown rice (unpolished rice varieties) were added as separate items as they vary in nutrient-content. Also, I observed that red rice was a staple food eaten by people from Mangalore and Kerala, while brown rice was eaten by people who are health-conscious or who have relatives at home with diabetes or who stay in rural areas. Rice vermicelli, rice flakes and puffed rice are similar in nutritive value and hence were coupled together as one item on the FFQ.

**Ragi foods:** Ragi (finger millet) vermicelli and ragi malt were added, they are perceived as easy to digest and useful to relieve the acidity common among pregnant woman.

**Vegetable sides:** To this section I added mushroom, green leafy curry and all types of gourd palyas. Drumstick is commonly consumed in curry, and sambhar (a lentil side dish) and was grouped separately. Beetroot, yam and tapioca were coupled together as one FFQ item because they are all root and tuber vegetables of similar nutrient-content.

**Ready to eat breakfast cereals:** A majority of Indians consume fresh, home-cooked breakfast and traditional foods like idly, dosa, poori, upma, seasoned rice and paratha, but ready to eat breakfast cereals are becoming more popular. Hence a separate section was added for these, including oats, wheat flakes, muesli, bran flakes, corn flakes (plain and with dry fruit) and chia seeds.

**Sweet snacks:** Popular branded sweet biscuits such as Parle-G, Glucose, Tiger, Marie and Gooday were added as one item. Branded salted and cream biscuits were added as two separate items. Popular sweet dishes like kesaribaath, obattu, barfi, jamun, jalebi, jangir, halwa, kheer, rasgulla, rasmalai, chum chum, champakali and malai sandwich were added. Regional names were used where applicable (for example, obattu is also known as holige in Kannada, boli in Tamil, bobatlu in Telugu and puranpoli in Marathi). To reduce the number of items, sweet dishes with similar nutritive value and ingredients were grouped together. For example, Gulab jamun, jalebi and jangir all contain refined wheat flour and sugar as the main ingredients, and all contain about 150 kilo calories per portion of about 40 grams. Similarly, ladoo and mysore pak contain chickpea flour/besan, sugar and ghee, with both containing approximately 250 kilo calories per portion of 45 grams. Bengali sweets like rasgulla, rasmalai, chum chum, champakali and malai sandwich were grouped together, as they are made of curdled milk and sugar, containing 300 kilo calories per portion of 68 grams. Western style desserts such as pudding, soufflé, custard and cheese cake were combined together as they all contain about 350 kilo calories and are usually consumed by urban women.

**Non vegetarian foods:** Non-vegetarian foods including chicken, fish and mutton in their various forms such as curry, sambhar, fry and cutlets were added. Dry fish was added separately. Seafood including prawn, crab and shell fish and organ meat including liver, brain and kidney were added separately. Egg, as omelette and in the boiled form, were added with and without the yolk (commonly not eaten for religious reasons or personal choice).

**Chutneys:** Coconut and groundnut chutneys were frequently consumed with dosa, idli, rice, chapathi, puri, upma and bhajji, and so were added to the FFQ.

**Sugar and jaggery:** Jaggery (a traditional concentrated product of sugar cane juice) was added, as it is used as a substitute for sugar, sometimes with tea, coffee, milk or by itself. Items originally on the Parthenon FFQ including sugar with idli, dosa, puri and chapathi were removed because these are usually consumed by children.

**Fast foods:** Fast food chains such as Burger King, KFC, McDonalds and Pizza Hut are now popular in India. Pizza and burger were categorised separately as vegetarian and non-vegetarian foods respectively.

**Savoury snacks:** Savoury snacks commonly available in bakeries, shops, and restaurants such as Gobi Manchurian, pakoda, bhajji, bonda, samosa, chips (potato/plantain), puff, chakli, murukku and kodbale were added. As for sweet snacks, similar ingredient and calorie items were grouped together to reduce the size of the FFQ. For example, pakoda, bhajji, bonda and samosa all contain chickpea flour, are deep fried in oil and contain about 300 kilo calories per serving. Urad vada was a separate FFQ item as it was made at home or bought from takeaway-restaurants, and frequently eaten for breakfast on weekends.

**Milk products:** Paneer (cottage cheese) tikka, curry, grated processed cheese, mozzarella and cheese spread were added under dairy products. These were not in the Mysore Parthenon FFQ but are commonly consumed in Bangalore.

**SUPPLEMENTARY SECTION 4: SUMMARY OF FOOD FREQUENCY QUESTIONNAIRE VALIDATION**

| **Statistical technique** | **Objective**  **(Test tool: FFQ**  **Reference tools: 24-hr recall & blood biomarkers)** | **Results** | **Facet of validation explored** |
| --- | --- | --- | --- |
| **Correlation** | Correlation between food-groups on the FFQ & 24-hr recall. | Daily staples (wholegrains, salads, fruit, dairy) showed good correlations.  Weekend foods – meat, fish, poultry showed poor correlations. | Relative validity |
| **Kappa statistic** | Frequency with which foods eaten at least 1/day on the FFQ were also seen on 24-hr recall. | Coffee, ragi ball, chapatti, rice, tea, fruit showed substantial to moderate agreement.  Lentils – poor agreement | Agreement |
| **Bland Altman plots** | Comparison of dietary patterns derived using the test tool and the 24 hour recall data | Dietary pattern 2 showed narrower limits of agreement (higher factor loadings for more number of staple foods). | Absolute validity and strength of association. |
| **Regression** | Association between intake (dietary micronutrient scores – FFQ) & status (blood biomarkers of folate, B12 & ferritin) | Dietary folate score was positively associated with plasma folate levels.  B12, ferritin – no association | Absolute validity |

The BANGLES FFQ was validated using a combination of four approaches against the reference tools (24-hour recall and blood biomarkers). The validation process was done post-hoc as we assessed a recall of the peri-conceptional diet at the time of recruitment, during early pregnancy. Correlation measured relative validity and kappa statistic, Bland-Altman plots and biomarkers measured absolute validity. Dietary data of 510 women from late pregnancy consisting of FFQ and a single 24-hour recall, both collected at the same visit, was used, for the correlation, Kappa and PCA methods followed by Bland-Altman plots.

Validation approach (D) Dietary nutrient intake versus status using biomarkers: Here, diet and blood data from 152 women who developed GDM and 152 age and BMI matched controls were used. In the early pregnancy FFQ (recalled peri-conceptional diet), the test variables used were weekly frequency of intake of food-groups rich in folate, B12 and iron.

The FFQ showed moderate to good correlation, percentage and limits of agreement with the reference tool (24-hour recall). Also the FFQ indicating ‘intake’ showed positive association with the ‘status’ of blood folate levels but not with the other blood biomarkers (plasma B12 and serum ferritin). The four approaches used explored different facets of validation:

Correlation –assessed relative validity of the test tool (FFQ) against the reference tool (24-hou

recall).

Percentage agreement (cross-classification) and Cohen’s Kappa statistic – assessed agreement for foods consumed and not consumed in both and either of the test (FFQ) and reference (24-hour) tools.

Regression – assessed absolute validity and strength of association where the FFQ as compared to a superior reference tool (blood biomarkers). This analysis found that a higher dietary folate score (intake) was associated with higher plasma folate levels (status). Dietary scores of iron and B12 intake were not associated with their blood statuses respectively, which may be explained by lower intake of iron & B12 rich foods.

Bland Altman plots – tested limits of agreement measuring absolute validity by comparing the dietary patterns from the FFQ and reference tool (24-hour recall), derived by Principal Component Analysis (PCA) method.

**Results**

**A). Correlation of food-groups constructed from the FFQ and 24-hour recall: Table 1** shows correlation coefficients (r) for 20 food-groups generated using the FFQ and the 24-hour recall. Frequently consumed foods including wholegrain cereals, salads and raw vegetables, fresh fruit, dried fruit and fruit juices, nuts and seeds, milk and dairy products, added sugar and SSB, coffee and nutrient-enriched beverage supplements showed good or moderate correlations. Milled cereals, fish and seafood, egg, poultry, meat, high salt products and visible fat showed poor correlations, as did pulses, cooked vegetables, cooked green leafy vegetables, potato containing dishes and potato palya (side dish).

**(B). Percentage of agreement (between FFQ and 24-hour recall) of commonly consumed foods using the Kappa statistic:** **Table 2** shows the percentage agreement between the FFQ and the 24-hour recall of weekly frequency of intake of 26 daily consumed food-items. Coffee and ragi ball showed substantial agreement while tea, milk, fresh fruit juice, green grapes, rice varieties and chapathi showed moderate agreement. Also buttermilk, tender coconut water, apple, big and small banana varieties, dates, raisins, walnuts, plain boiled white rice, curd rice, raw cucumber, bread and biscuits showed fair agreement. Lastly, pomegranate and guava showed slight agreement while lentil sides showed poor agreement.

**Table 1: Correlation between the 21 food-groups constructed using the 24-hour recall and the FFQ (n = 510)**

| **Food-group** | **r** |
| --- | --- |
| Milled cereals and products | 0.22** |
| Whole grain cereals | 0.67** |
| Pulse and lentil preparations | 0.07** |
| Salads and raw vegetables | 0.30** |
| Cooked vegetables | 0.10* |
| Cooked green leafy vegetables | 0.18** |
| Fresh fruit, dried fruit and fruit juices | 0.36** |
| Nuts and seeds | 0.32** |
| Milk and dairy products | 0.54 |
| Fried and fast food | 0.16 |
| High sugar products, added sugar and SSB ^a^ | 0.48** |
| Potato containing dishes | 0.17** |
| Potato palya (potato side dish) | 0.11* |
| Coffee | 0.65** |
| Nutrient enriched beverage supplements | 0.35** |
| Fish and seafood | 0.23** |
| Egg and egg preparations | 0.23** |
| Poultry | 0.25** |
| Meat | 0.28** |
| High salt products | 0.27 |
| Visible fat | 0.23** |
| Interpretation of ‘r’: r>0.3 indicates good correlation; r>0.2 indicates moderate correlation & r<0.2 indicates poor correlations.  * means p<0.05; ** means p<0.01  Grey coloured boxes indicate Pearson’s correlation co-efficient. | |

**Table 2: Percentage agreement between the FFQ and 24-hour recall in terms of frequency of intake of 26 foods (n=510)**

| **Foods** | **N (%) of agreement** | | **N (%) of disagreement** | | **Cohen’s Kappa**  **statistic** | **Level**  **of agreement**  **based on Kappa statistic^a^** | |
| --- | --- | --- | --- | --- | --- | --- | --- |
|  | **N (%) reported in FFQ and 24-hour** | **N (%) not reported in FFQ or 24-hour** | **N(%) FFQ only** | **N(%) reported in 24-hour only** |  |  |  |
| **Beverages:** | | | | | | |  |
| Tea | 110 (74.8) | 315 (86.8) | 37 (25.2) | 48 (13.2) | 0.60** | Moderate | |
| Coffee | 83 (71.6) | 371 (94.2) | 33 (28.4) | 23 (5.8) | 0.68** | Substantial | |
| Milk | 312 (95.1) | 88 (48.4) | 16 (4.9) | 94 (31.6) | 0.48** | Moderate | |
| Buttermilk | 41 (57.7) | 368 (83.8) | 30 (42.3) | 71 (16.2) | 0.34** | Fair | |
| Tender coconut water | 15 (44.1) | 445 (93.5) | 19 (55.9) | 31 (6.5) | 0.32** | Fair | |
| Fresh fruit juice | 23 (52.3) | 436 (93.6) | 21 (47.7) | 30 (6.4) | 0.42** | Moderate | |
| **Fruits:** | | | | | | |  |
| Apple | 84 (66.7) | 272 (70.8) | 42 (33.3) | 112 (29.2) | 0.32** | Fair | |
| Big banana (Robusta) | 22 (45.8) | 421 (91.1) | 26 (54.2) | 41 (8.9) | 0.32** | Fair | |
| Small banana (Yelakki type) | 44 (62.9) | 341 (77.5) | 99 (22.5) | 26 (37.1) | 0.28** | Fair | |
| Pomegranate | 6 (33.3) | 455 (92.5) | 12 (66.7) | 37 (7.5) | 0.16** | Slight | |
| Guava | 4 (22.2) | 477 (97.0) | 14 (77.8) | 15 (3.0) | 0.19** | Slight | |
| Green grapes | 10 (52.6) | 483 (98.4) | 9 (47.4) | 8 (1.6) | 0.52** | Moderate | |
| **Dried fruits & nuts:** | | | | | | |  |
| Dates | 38 (97.4) | 331 (70.3) | 1 (2.6) | 140 (29.7) | 0.26** | Fair | |
| Raisins | 32 (84.2) | 345 (73.1) | 6 (15.8) | 30 (6.1) | 0.23** | Fair | |
| Walnuts | 11 (57.9) | 461 (93.9) | 8 (42.1) | 30 (6.1) | 0.33** | Fair | |
| **Cereal staples:** | | | | | |  | |
| Plain boiled white rice | 434 (97.7) | 11 (16.7) | 10 (2.3) | 55 (83.3) | 0.20** | Fair | |
| Curd rice | 10 (40.0) | 459 (94.6) | 26 (5.4) | 15 (80.0) | 0.29** | Fair | |
| Rice varieties* | 470 (98.1) | 8 (25.8) | 9 (1,9) | 23 (74.2) | 0.30** | Moderate | |
| Chapathi | 85 (48.3) | 300 (89.8) | 91 (51.7) | 34 (10.2) | 0.41** | Moderate | |
| Ragiball | 165 (91.2) | 272 (82.7) | 16 (8.8) | 57(17.3) | 0.70** | Substantial | |
| Ragi ganji | 11 (57.9) | 481 (98.0) | 8 (42.1) | 10 (2.0) | 0.53** | Moderate | |
| **Vegetables & lentil sides:** | | | | | | |  |
| Cooked vegetable palya sides | 177 (61.5) | 97 (43.7) | 111 (38.5) | 125 (56.3) | 0.05 | Equivalent to chance | |
| Lentil sides score | 263 (65.8) | 43 (39.1) | 67 (60.9) | 137 (34.3) | 0.03 | Equivalent to chance | |
| Raw cucumber | 19 (63.3) | 426 (88.8) | 11 (36.7) | 54 (11.3) | 0.31** | Fair | |
| **Snacks:** | | | | | | |  |
| Biscuits | 53 (51.5) | 338 (83.0) | 50 (48.5) | 69 (17.0) | 0.32** | Fair | |
| White bread | 14 (29.8) | 442 (95.5) | 33 (70.2) | 21 (4.5) | 0.29** | Fair | |

**Summary of the FFQ validation procedure:** The BANGLES FFQ is a valid method to assess the dietary frequency of intake of most food-groups. These include milled cereal products (including rice preparations), wholegrain cereals (including individual wholegrain cereal components such as unpolished rice, chapathi and millet preparations such as ragi ball, ragi ganji), raw vegetables, fruits, nuts and seeds, milk and dairy products, high sugar products, coffee, fish, egg, poultry, meat and visible fat sweet snacks including biscuits. It makes sense that these foods are accurately reported as it is often easier for respondents to remember and report foods that they frequently consume. In addition, foods that are eaten daily are much more likely to be reported on the 24-hour recall, whereas those eaten less frequently e.g. pulses and cooked vegetables will not necessarily be reported during the 24-hour recall. Based on this analysis, it is considered as an appropriate tool to assess dietary intake among reproductive-aged women in South India.

**SUPPLEMENTARY SECTION 5: 55 FOOD-GROUPS, THEIR COMPONENTS AND MODIFICATIONS TO CONSTRUCT THE 68 FOOD-GROUPS**

| **Food-groups** | **Food-group components** | **Change in the new 68 food- groups** |
| --- | --- | --- |
| **Commonly consumed beverages** | Tea | Grouped together as one group |
|  | Coffee |  |
| **Milk** | Normal cows milk (as plain milk / \|Horlicks etc.) | Retained |
|  | Skimmed cows milk (as plain milk / Horlicks etc.) |  |
| **Fermented dairy products** | Buttermilk | Retained |
|  | Raitha (any vegetable raita) |  |
| **Flavoured sweetened nutrient supplements** | Horlicks, Complan, Boost, Bournvita | Retained |
|  | Pro PL |  |
|  | Baby and me |  |
|  | Pristine balance PL |  |
|  | Nutri right mom |  |
|  | GRD Smart |  |
|  | Protovic DF |  |
| **Flavoured sweetened beverages** | Glucon d powder | Retained |
|  | Hersheys syrup / choco syrup |  |
|  | Badam milk |  |
|  | Sugarcane juice |  |
|  | Packaged fruit juice(real, tropicana etc.) |  |
|  | Coke/ pepsi / sprite / mirinda / fanta or other fizzy drink |  |
|  | Frooti / Maaza / Slice / \|Nimbooz / Appy fizz |  |
|  | Squashes / syrups (tang/kisan/ rasna/ roohafza/lemonade) |  |
| **Therapeutic beverages** | Electoral | Retained |
|  | Tender coconut water |  |
| **Soups** | Fresh vegetable soups | Retained |
|  | Rasam |  |
|  | Herbal drinks |  |
|  | Fresh non - veg soups |  |
| **Fresh fruit juices** | Fresh fruit juice specify fruit: | Retained |
| **Low calorie fruits** | Apple | Not retained.  All fruits combined with commonly consumed fruits kept separate such as apple, sweet lime, banana and pomegranate. |
|  | Sweet lime |  |
|  | Guava |  |
|  | Pomegranate |  |
|  | Musk melon |  |
|  | Pear |  |
|  | Papaya |  |
| **High calorie fruits** | Banana (pacche/robusta/ nendra - kerala type) |  |
|  | Banana (yelakki) |  |
|  | Sapota |  |
|  | Grapes (green) |  |
|  | Pineapple |  |
|  | Avocado |  |
| **Mixed fruit salad** | Mixed fruit salad |  |
| **Dried fruits** | Dates | Grouped separately |
|  | Raisins |  |
|  | Figs | Grouped separately |
|  | Prunes |  |
|  | Apricots |  |
| **Nuts and seeds + preparations** | Almonds | Grouped separately |
|  | Cashewnuts | Grouped separately |
|  | Pistachios |  |
|  | Sunflower seeds |  |
|  | Pumpkin seeds |  |
|  | Flax seeds |  |
|  | Groundnuts |  |
|  | Walnuts |  |
|  | Chia seeds |  |
|  | Coconut chutney | Grouped separately |
|  | Groundnut chutney |  |
|  | Chikki | Grouped with nuts |
| **Polished rice preparations** | Plain/boiled rice | Both separated as individual groups since both were commonly consumed and eaten in different combinations |
|  | Colour / seasoned rice (tamarind / lemon / ghee / tomato) |  |
| **Unpolished rice** | Red rice | Retained |
|  | Brown rice |  |
| **Rice and dal preparations** | Rice with dal / sambhar | Kept separate  Combined with colour rice  Kept separate |
|  | Vegetable bath / bisi bele bath / veg khichdi |  |
|  | Idly |  |
| **Rice and greens preparations** | Rice with glv specify glv: | Retained |
| **Curd rice** | Curd rice | Retained |
| **Semolina/beaten rice and vegetable preparation** | Rice vermicelli / avalakki/ puffed rice (mandakki) | Retained |
|  | Uppuma |  |
| **Fried cereal and pulse preparations** | Plain dosa | Dosas kept separate |
|  | Masala dosa |  |
|  | Poori | Poori and parantha combined |
|  | Paratha |  |
|  | Urad vada | Urad vada and chilas  combined |
|  | Chilas / adai / pesarattu |  |
| **Whole grain breads** | Chapathi | Chapathi & broken wheat kept separate |
|  | Sliced brown bread | Moved into another group of western breads |
|  | Broken wheat / dalia |  |
| **Refined cereal preparations** | Rice roti | Combined as all are traditional rice dishes |
|  | Rice puttu/kadabu |  |
|  | Sweet bun / kara bun | Separately grouped as western breads |
|  | Sliced white bread |  |
|  | Pasta / macaroni |  |
|  | Spaghetti / noodles |  |
| **Ready to eat breakfast cereals** | Wheat flakes | Retained |
|  | Oat + milk |  |
|  | Oats + water |  |
|  | Muesli |  |
|  | All bran flakes |  |
|  | Corn flakes (plain) |  |
|  | Corn flakes (with dry fruit) |  |
| **Millet preparations** | Ragi roti | Retained |
|  | Ragi dosa |  |
|  | Ragi malt |  |
|  | Ragi vermicelli |  |
| **Ragi ball** | Ragi ball | Retained |
| **Whole and split pulse preparations** | Legume based dhal (green gram / bengal gram/ channa/rajma) | Retained |
|  | Split dal based dhal(moong/ toor/ masoor) |  |
|  | Mix veg sambhar |  |
|  | Kosambari | Except Kosambari separated as it is a traditional salad |
| **Roots and tuber sides** | Potato palya | Retained |
|  | Carrot palya |  |
|  | Beetroot, radish, yam, tapioca palya |  |
| **Other vegetable sides** | Ladysfinger palya | Retained |
|  | Chow chow, pumpkin, gourds |  |
|  | Capsicum palya |  |
|  | Drumstick palya |  |
|  | Brinjal palya |  |
|  | Beans playa |  |
|  | Mushroom palya |  |
|  | Multi vegetable saagu |  |
| **Green leafy vegetable sides** | Glv / soppu sambhar | Traditional GLV sides kept separate as a group. |
|  | Glv / soppu palya |  |
|  | Cabbage / knol khol palya | Other GLV sides as a separate group. |
|  | Cauliflower palya |  |
| **Raw vegetables** | Cucumber (without skin) | Retained |
|  | Tomato |  |
|  | Onion |  |
|  | Carrot |  |
|  | Radish |  |
|  | Runner beans |  |
| **Chicken preparations** | Chicken biriyani | Grouped separately |
|  | Chicken fry (roasted/grilled) | Grouped separately |
|  | Chicken curry |  |
| **Fish preparations** | Fish fry | Retained |
|  | Fish curry |  |
|  | Fish cutlet |  |
|  | Dried fish / dried seafood |  |
|  | Prawn, crab, shellfish |  |
| **Red meat preparations** | Lamb, beef, pork- fry | Retained |
|  | Lamb, beef, pork - cutlet |  |
|  | Lamb, beef, pork - curry |  |
|  | Liver, brain, kidney |  |
|  | Keema curry |  |
|  | Ham / salami / bacon |  |
|  | Mutton biriyani |  |
| **Egg preparations** | Egg – boiled (with yolk) | Grouped separately |
|  | Egg biriyani | Grouped separately |
|  | Egg - boiled (without yolk) |  |
|  | Egg burchi, curry |  |
|  | Omelette with yolk |  |
|  | Omelette without yolk |  |
| **High salt products** | Pickle (lemon / mango) | Grouped as traditional condiments with chutneys. |
|  | Papad/ sandige (home fried) |  |
|  | Chutney powders |  |
|  | Ketchup | Grouped separately as western condiments |
|  | Ready to make soups |  |
| **High sugar additives** | Jam | Retained |
|  | Honey |  |
|  | Sugar in fruit juice |  |
|  | Sugar in tea |  |
|  | Sugar in coffee |  |
|  | Sugar in milk |  |
|  | Jaggery |  |
| **Medium calories fried savoury snacks** | Namkeen/mixtures/savouries | Retained |
|  | Chakli/murukku/kodbale |  |
| **High calories fried savoury snacks** | Pakoda/bonda/bhajji/samosa | Retained |
|  | Babycorn / gobi manchurian |  |
|  | Chips (potato/plantain)french fries |  |
| **Puff pastry snacks** | Puff | Retained |
| **Chocolates** | Chocolate*specify brand: | Retained |
|  | Toffee/ candy |  |
| **Cakes** | Cake without cream | Retained |
|  | Cake with cream / pastry |  |
| **Biscuits** | Sweet biscuits (eg: parle g / glucose / tiger / marie / goodday) | Retained |
|  | Salted biscuitseg: monaco / 50 - 50 / krackjack / maska chaska |  |
|  | Cream biscuitseg: dream cream, milky bite, jim jam, bourbon, britannia pure ma |  |
| **Cereal based sweet preparations** | Kesaribaath | Retained, named as traditional homemade sweets with Kheer. |
|  | Obbattu/ holige / boli/ bobatlu/ puranpoli |  |
| **Fried sweet preparations** | Barfi /pedha | Retained, named as shop-bought Indian sweets. |
|  | Gulab jamoon, jalebi. jangir |  |
|  | Laddoo, mysore pak |  |
|  | Halwa (dumroot/carrot) |  |
| **Milk based sweet preparations** | Kheer / payasam | Grouped with traditional homemade sweets. |
|  | Bengali sweets - rasgulla / rasmalai / chum chum / champakali/ malai sandwich | Merged with shop-bought Indian sweets. |
|  | Pudding/custard/souffle/cheese cake |  |
| **Chats** | Masala puri, pani puri, bhel puri etc. | Retained |
| **Fast foods** | Veg pizza | Retained |
|  | Non veg pizza |  |
|  | Veg burger |  |
|  | Non veg burger |  |
| **Curd/yogurt** | Curd | Retained |
| **Butter and clarified butter** | Butter | Each grouped separately. |
|  | Ghee on the plate/ table |  |
| **Cheese and paneer** | Cheese as: (grated cheddar/mozarella, spread and all types) | Retained |
|  | Paneer curry(paneer butter masala/kadai paneer) |  |
|  | Paneer tikka |  |
| **Ice creams & Milkshakes** | Icecreams*include cup & stick icecreams | Retained |
|  | Milkshakes |  |
| **Low calories seasonal fruits** | Orange | All seasonal fruits combined. |
|  | Purple grapes |  |
|  | Watermelon |  |
|  | Litchi |  |
|  | Amla |  |
|  | Jamun |  |
|  | Custard apple |  |
| **High calories seasonal fruits** | Raw mango |  |
|  | Ripe mango |  |
|  | Green peas (palya / sambhar / snacks / upma) |  |
|  | Plum |  |
|  | Jackfruit |  |
| **Seasonal green beans** | Avrekalu | Retained |
|  | Green peas |  |
| **Sweet corn** | Boiled / roasted corn | Combined into cooked vegetables |

**SUPPLEMENTARY SECTION 6: PCA OF ALL FOODS**

PCA of all foods consumed (205 foods) was carried out to observe which food discriminates a particular diet pattern, this information may be lost when PCA is done on pre-grouped foods.

**The figure 6a below,** shows the scree plots for the PCA of 205 foods. Using this, I decided to retain the first three output components that represent the three diet patterns. The scree plot break, where the line becomes more horizontal, is often used to decide which components to include. We can see the break in the scree plot starts to occur after the 3^rd^ point. The Kaiser-Meyer-Olkin test (KMO) measure of sampling adequacy was 0.64 for the PCA of 205 foods, indicating that the sample size for the PCA was adequate. The first three components explained about 9.98 percent of variance in the sample (**table 3a**).

**Figure 6a: Scree plot of PCA from 205 foods consumed by women in the FFQ (n=785)**


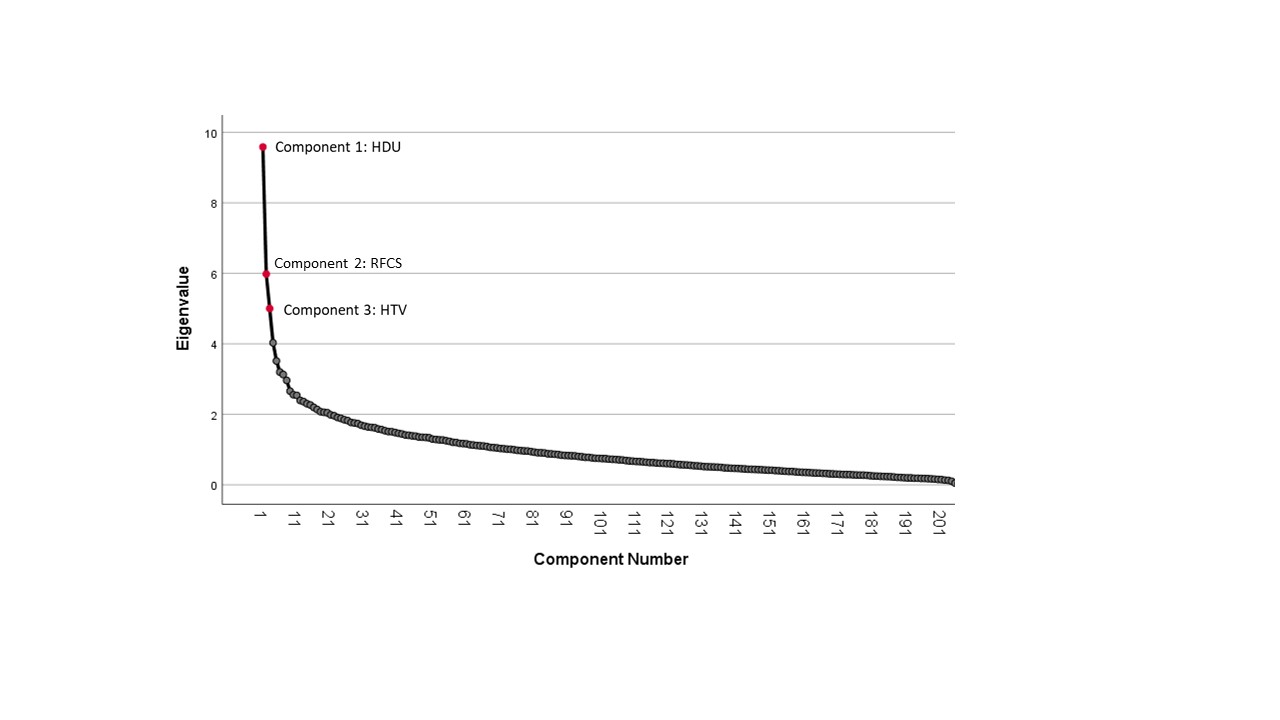


| **Table 6a: Total variance explained by PCA of 205 foods (n=785)** | |
| --- | --- |
| **Component** | **Cumulative % of variance** |
| 1 | 4.65 |
| 2 | 7.56 |
| 3 | 9.98 |

The three diet patterns were distinct and based on their characteristics (**table 3b** shows that the PCA of 205 foods), were named as follows:

Pattern 1: High-diversity, urban

Pattern 2: Rice-fried snacks-chicken-sweets

Pattern 3: Healthy, traditional vegetarian

The purpose of doing a PCA of 205 foods was to identify pattern discriminating foods, which are foods with high factor loadings in each pattern, and which stood out in a particular pattern. This enabled me to create 68 food- groups, using a cut-off of 0.2 for The PCA of 205 foods served as a good template to devise grouping of foods, to actually see how foods appear distinctly in each pattern and get detailed elements of each diet pattern.

**Table 6b: Unrotated PCA of 205 FFQ foods showing factor loadings of the first 3 diet patterns (n=785)**

| **Foods in the FFQ** | **Diet patterns** | | |
| --- | --- | --- | --- |
|  | **1**  **HDU** | **2**  **RFCS** | **3**  **HTV** |
| **Beverages:** | | | |
| Tea | 0.01 | 0.21 | -0.21 |
| Coffee | -0.09 | 0.14 | 0.09 |
| Normal cow’s milk (as plain milk / Horlicks etc.) | 0.17 | -0.06 | 0.24 |
| Skimmed cow’s milk (as plain milk / Horlicks etc.) | 0.09 | -0.10 | 0.01 |
| Buttermilk | -0.03 | 0.15 | 0.36 |
| Horlicks, Complan, Boost & Bournvita | 0.14 | -0.04 | 0.14 |
| Pro PL | -0.08 | -0.01 | 0.00 |
| Baby & me | 0.03 | 0.04 | 0.08 |
| Pristine Balance PL | 0.01 | 0.03 | 0.01 |
| Nutri-right Mom | -0.01 | -0.05 | 0.01 |
| GRD Smart | -0.02 | -0.01 | 0.03 |
| Protovic DF | -0.01 | 0.03 | 0.08 |
| Glucon D | 0.03 | 0.05 | 0.08 |
| Hersheys chocolate syrup | 0.06 | 0.09 | -0.03 |
| Badam milk | 0.13 | 0.10 | 0.11 |
| Electoral | 0.01 | 0.08 | 0.04 |
| Tender coconut water | 0.21 | 0.07 | 0.21 |
| Sugarcane juice | 0.17 | 0.17 | -0.01 |
| Packaged fruit juice (Real, tropicana etc.) | 0.24 | 0.05 | -0.17 |
| Fresh vegetable soups | 0.23 | -0.05 | 0.06 |
| Fresh non - veg soups | 0.20 | 0.07 | -0.12 |
| Ready to make soups | 0.11 | -0.03 | -0.05 |
| Carbonated beverages | 0.27 | 0.21 | -0.23 |
| Frooti / maaza / slice / nimbooz / appy fizz | 0.14 | 0.20 | -0.09 |
| Squashes / syrups (tang/kisan/ rasna/ roohafza/lemonade) | 0.09 | 0.07 | -0.11 |
| Fresh fruit juice specify fruit | 0.30 | 0.03 | 0.13 |
| Milkshakes | 0.19 | 0.07 | -0.08 |
| Herbal drinks | 0.15 | -0.05 | 0.05 |
|  | | | |
| Apple | 0.22 | 0.12 | 0.26 |
| Banana (pacche/robusta) kerala type) | 0.15 | 0.11 | 0.01 |
| **Banana (yelakki)** | 0.12 | 0.26 | 0.18 |
| Sweetlime | 0.15 | 0.15 | 0.22 |
| Guava | 0.12 | 0.06 | 0.09 |
| Pomegranate | 0.25 | 0.16 | 0.25 |
| Sapota | 0.14 | 0.09 | 0.14 |
| Grapes (green) | 0.20 | 0.06 | 0.14 |
| Musk melon | 0.27 | -0.10 | 0.12 |
| Pear | 0.28 | -0.11 | 0.06 |
| Papaya | 0.32 | -0.09 | 0.14 |
| Pineapple | 0.18 | 0.12 | 0.02 |
| Mixed fruit salad | 0.13 | 0.01 | -0.06 |
| Avocado | 0.22 | -0.23 | 0.06 |
| **Dried fruits and nuts:** | | | |
| Dates | 0.28 | 0.16 | 0.24 |
| Raisins | 0.28 | 0.09 | 0.29 |
| Figs | 0.25 | -0.06 | 0.09 |
| Prunes | 0.20 | -0.19 | 0.10 |
| Cashew nut | 0.27 | 0.11 | 0.21 |
| Almond | 0.48 | -0.05 | 0.17 |
| Pistachios | 0.26 | 0.07 | 0.09 |
| Apricots | 0.21 | -0.01 | 0.11 |
| Sunflower seeds | 0.04 | -0.04 | 0.04 |
| Pumpkin seeds | 0.14 | -0.16 | 0.04 |
| Flaxseeds | -0.06 | -0.04 | 0.01 |
| Walnuts | 0.28 | -0.37 | 0.14 |
| Groundnuts | 0.09 | 0.15 | 0.17 |
| **Rice and wheat foods:** | | | |
| Plain/boiled rice | -0.33 | 0.44 | 0.11 |
| Red rice | 0.02 | -0.09 | -0.09 |
| Brown rice | 0.02 | -0.09 | 0.07 |
| Rice with dal / sambhar | -0.14 | 0.31 | 0.13 |
| Rice with glv specify glv: | -0.09 | 0.30 | 0.32 |
| Spice Seasoned/ flavoured rice (tamarind / lemon / ghee / tomato) | -0.08 | 0.45 | 0.22 |
| Vegetable bath / bisi bele bath / veg khichdi | 0.14 | 0.22 | 0.23 |
| Curd rice | 0.09 | -0.02 | 0.21 |
| Rice vermicelli / avalakki/ puffed rice (mandakki) | 0.24 | -0.14 | 0.14 |
| Idly | 0.16 | 0.12 | -0.14 |
| Plain dosa | 0.11 | 0.14 | -0.14 |
| Masala dosa | 0.28 | 0.13 | -0.07 |
| Rice roti | 0.10 | 0.10 | 0.24 |
| Rice puttu/ kadabu | 0.04 | -0.02 | -0.16 |
| **Chapathi** | 0.38 | -0.29 | -0.01 |
| Poori | 0.22 | 0.23 | -0.05 |
| Parantha | 0.30 | -0.25 | -0.08 |
| Sweet bun / kara bun | 0.06 | 0.16 | -0.12 |
| Sliced white bread | 0.11 | 0.30 | 0.02 |
| Sliced brown bread | 0.31 | -0.34 | -0.08 |
| Uppuma | 0.16 | 0.08 | 0.21 |
| Broken wheat / dalia | 0.15 | -0.15 | 0.08 |
| Wheat flakes | 0.13 | -0.12 | 0.02 |
| Pasta / macaroni | 0.33 | -0.10 | -0.22 |
| Spaghetti / noodles | 0.27 | 0.01 | -0.22 |
| **Ragi foods:** | | | |
| Ragi roti | 0.04 | 0.09 | 0.28 |
| **Ragi ball** | -0.33 | 0.30 | 0.34 |
| Ragi dosa | 0.05 | 0.03 | 0.22 |
| Ragi Malt / Malt / Ganji | 0.01 | 0.08 | 0.09 |
| Ragi vermicelli | 0.05 | 0.00 | 0.06 |
| **Ready to eat breakfast cereals:** | | | |
| Oat + milk | 0.15 | -0.16 | 0.01 |
| Oats + water | 0.20 | -0.17 | 0.03 |
| Muesli | 0.26 | -0.22 | 0.04 |
| Corn flakes (plain) | 0.10 | -0.06 | 0.01 |
| Corn flakes (with dryfruit) | 0.32 | -0.19 | 0.01 |
| All bran flakes | 0.20 | 0.02 | 0.03 |
| Chia seeds | 0.25 | -0.20 | 0.15 |
| **Pulse and lentil foods:** | | | |
| Urad vada | 0.21 | 0.16 | -0.15 |
| Chilas / adai / pesarattu | 0.22 | -0.11 | -0.01 |
| **Pulse and vegetable sides:** | | | |
| Legume based dhal | 0.12 | 0.13 | 0.22 |
| Split lentil based dhal | 0.08 | -0.14 | 0.11 |
| Potato palya | 0.26 | 0.14 | -0.23 |
| Carrot palya | 0.26 | 0.04 | 0.14 |
| Mixed veg sambhar | -0.12 | 0.24 | 0.14 |
| Glv / soppu sambhar | -0.11 | 0.36 | 0.34 |
| Beetroot, radish, yam, tapioca palya | 0.24 | 0.18 | 0.16 |
| Ladysfinger palya | 0.30 | 0.01 | -0.03 |
| Cabbage / knol khol palya | 0.27 | -0.03 | -0.01 |
| Cauliflower palya | 0.48 | -0.03 | -0.21 |
| Chow chow, pumpkin, gourds | 0.29 | -0.08 | 0.11 |
| Capsicum palya | 0.34 | -0.06 | 0.12 |
| Drumstick palya | 0.14 | 0.18 | 0.00 |
| Brinjal palya | 0.29 | 0.08 | -0.07 |
| Beans playa | 0.33 | 0.10 | 0.08 |
| Mushroom palya | 0.34 | -0.03 | -0.08 |
| Glv / soppu palya | 0.16 | 0.25 | 0.23 |
| Multi vegetable saagu | 0.24 | 0.05 | 0.10 |
| Rasam | 0.05 | 0.20 | 0.09 |
| **Raw vegetables and salads:** | | | |
| Cucumber (without skin) | 0.33 | 0.03 | 0.31 |
| Tomato | 0.40 | -0.12 | 0.10 |
| Onion | 0.22 | -0.13 | -0.03 |
| Carrot | 0.33 | 0.03 | 0.31 |
| Radish | 0.21 | 0.00 | 0.10 |
| Kosambari | 0.11 | 0.10 | 0.36 |
| Runner beans | 0.04 | 0.18 | 0.18 |
| Raw Greens | 0.27 | -0.11 | 0.13 |
| **Non-vegetarian preparations:** | | | |
| Chicken biriyani | 0.14 | 0.38 | -0.30 |
| Chicken fry | 0.22 | 0.30 | -0.36 |
| Chicken curry/ sambhar | 0.14 | 0.33 | -0.26 |
| Fish fry | 0.28 | 0.07 | -0.29 |
| Fish curry | 0.19 | -0.01 | -0.22 |
| Fish cutlet | 0.13 | -0.02 | -0.25 |
| Dried fish / dried seafood | 0.12 | 0.10 | -0.17 |
| Prawn, crab, shellfish | 0.34 | -0.07 | -0.21 |
| Lamb, beef, pork- fry | 0.10 | 0.18 | -0.24 |
| Lamb, beef, pork - cutlet | 0.05 | 0.00 | -0.06 |
| Lamb, beef, pork - curry | 0.19 | 0.20 | -0.29 |
| Liver, brain, kidney | 0.19 | 0.16 | -0.21 |
| Keema curry | 0.36 | 0.09 | -0.23 |
| Mutton biriyani | 0.25 | 0.19 | -0.26 |
| Egg biriyani | 0.07 | 0.27 | 0.00 |
| Egg – boiled (with yolk) | 0.12 | 0.27 | -0.06 |
| Egg - boiled (without yolk) | 0.06 | -0.10 | 0.05 |
| Egg burchi, curry | 0.27 | 0.09 | -0.22 |
| Omelette with yolk | 0.21 | 0.26 | -0.20 |
| Omelette without yolk | 0.01 | 0.06 | 0.11 |
| **Condiments:** | | | |
| Pickle (lemon / mango) | 0.12 | 0.28 | 0.02 |
| Coconut chutney | 0.18 | 0.16 | -0.01 |
| Groundnut chutney | 0.02 | 0.17 | 0.03 |
| Tomato Ketchup | 0.31 | -0.10 | -0.12 |
| Jam | 0.21 | 0.19 | 0.02 |
| Honey | 0.29 | -0.24 | 0.04 |
| Other chutneys and powders | 0.10 | -0.22 | -0.04 |
| **Added sugar and jaggery:** | | | |
| Sugar in fruit juice | 0.16 | 0.18 | 0.05 |
| Sugar in tea | -0.04 | 0.22 | -0.20 |
| Sugar in coffee | -0.12 | 0.17 | 0.09 |
| Sugar in milk | 0.10 | 0.04 | 0.18 |
| Jaggery | 0.12 | -0.19 | 0.14 |
| **Savoury (salted) snacks:** | | | |
| Papad/ sandige (home fried) | 0.19 | 0.25 | 0.01 |
| Namkeen/mixtures/savouries | 0.15 | 0.24 | -0.04 |
| Chakli/murukku/kodbale | 0.11 | 0.34 | 0.04 |
| Pakoda/bonda/bhajji/samosa | 0.07 | 0.28 | -0.14 |
| Babycorn / gobi manchurian | 0.21 | 0.31 | -0.04 |
| Boiled / roasted corn | 0.23 | 0.12 | 0.08 |
| Chips (potato/plantain/fries) | 0.20 | 0.28 | -0.18 |
| Puff | 0.14 | 0.34 | -0.07 |
| Chats (masala puri, pani puri, bhel puri) | 0.22 | 0.23 | -0.10 |
| **Sweet snacks:** | | | |
| Chocolate*specify brand: | 0.35 | 0.12 | -0.13 |
| Toffee/ candy | 0.15 | 0.07 | -0.15 |
| Cake without cream | 0.20 | 0.17 | -0.03 |
| Cake with cream / pastry | 0.17 | 0.26 | -0.08 |
| Sweet biscuits | 0.13 | 0.19 | 0.07 |
| Salted biscuits | 0.16 | 0.10 | -0.15 |
| Cream biscuits | 0.18 | 0.10 | -0.04 |
| Kesaribaath | 0.12 | 0.17 | 0.00 |
| Obbattu/holige | -0.02 | 0.08 | 0.07 |
| Barfi /pedha | 0.16 | 0.11 | 0.00 |
| Gulab jamoon, jalebi. jangir | 0.14 | 0.20 | -0.08 |
| Chikki | 0.08 | -0.01 | 0.01 |
| Laddoo, Mysore pak | 0.14 | 0.12 | -0.05 |
| Halwa (dumroot/carrot) | 0.24 | 0.04 | -0.03 |
| Kheer/ payasam | 0.01 | 0.03 | 0.04 |
| Bengali sweets | 0.24 | 0.08 | -0.12 |
| Pudding/custard/cheese cake | 0.19 | -0.05 | -0.18 |
| **Fast food:** | | | |
| Veg pizza | 0.31 | -0.18 | 0.02 |
| Non veg pizza | 0.25 | 0.00 | -0.39 |
| Veg burger | 0.22 | -0.08 | -0.07 |
| Non veg burger | 0.16 | 0.02 | -0.32 |
| **Milk products:** | | | |
| Curd | 0.17 | -0.02 | 0.27 |
| Raitha (any vegetable raita) | 0.17 | 0.14 | 0.02 |
| Butter | 0.26 | 0.01 | -0.09 |
| Ghee on the plate/ table | 0.21 | 0.00 | 0.23 |
| Cheese (all types) | 0.32 | -0.23 | -0.07 |
| Paneer curry | 0.50 | -0.35 | -0.01 |
| Paneer tikka | 0.12 | -0.04 | 0.00 |
| Icecreams (all types) | 0.32 | 0.18 | -0.13 |
| **Seasonal foods**: | | | |
| Raw mango | 0.06 | 0.26 | 0.14 |
| Ripe mango | 0.22 | 0.01 | 0.10 |
| Jackfruit | 0.11 | 0.14 | 0.23 |
| Avrekalu (green beans) | -0.07 | 0.22 | 0.18 |
| Orange | 0.34 | 0.00 | 0.27 |
| Green peas | 0.37 | -0.17 | 0.07 |
| Purple grapes | 0.18 | 0.07 | 0.01 |
| Watermelon | 0.34 | 0.05 | 0.08 |
| Litchi | 0.40 | -0.20 | 0.08 |
| Amla | 0.17 | 0.06 | 0.12 |
| Jamun | 0.15 | 0.04 | 0.06 |
| Plum | 0.29 | -0.07 | -0.07 |
| Custard apple | 0.23 | -0.01 | 0.12 |
| Strawberry | 0.48 | -0.13 | 0.05 |
| **Lighter shadings: positive factor loadings >0.2; Darker shadings: negative factor loadings <-0.2** | | | |

**SUPPLEMENTARY SECTION 7**

**Figure: Scree plot from the PCA of the 68 food-groups (n=785)**


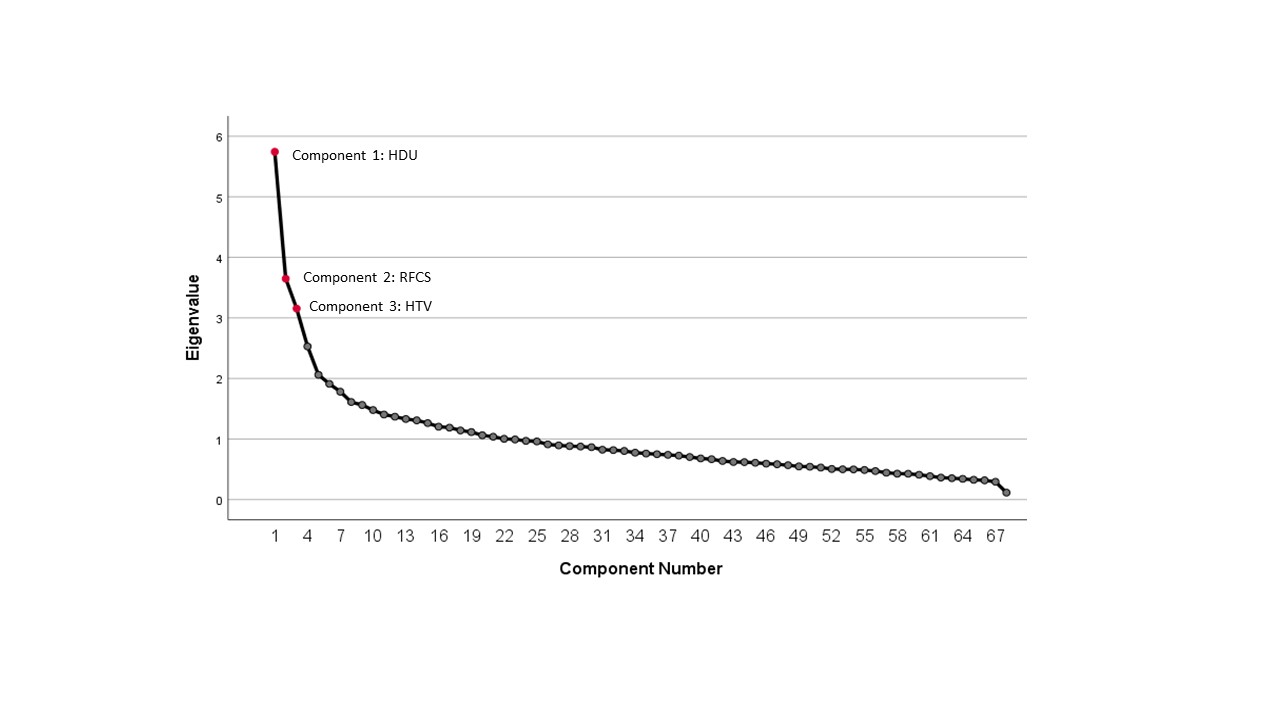


**SUPPLEMENTARY SECTION 8**

| **Table 3: Medians and IQR for weekly frequencies of intake of key foods according to quintiles of diet patterns scores (n=785)** | | | | | |
| --- | --- | --- | --- | --- | --- |
| **Key foods exemplifying the diet pattern** | **Quintiles of women’s diet pattern scores**  **(units: weekly frequency of intake of foods)** | | | | |
|  | **1** | **2** | **3** | **4** | **5** |
| **High-diversity, urban** | | | | | |
| Chapathi | 1.0  (0.5, 2.0) | 2.0  (1.0, 4.0) | 3.0  (1.0, 7.0) | 3.5  (2.0, 7.0) | 7.0  (2.0, 7.0) |
| Flavoured sweetened beverages | 0.25  (0.0, 1.0) | 0.5  (0.0, 1.2) | 0.7  (0.2, 2.0) | 1.0  (0.1, 2.3) | 2.0  (0.7, 5.1) |
| Other vegetables sides ^a^ | 1.25  (0.5, 2.0) | 2.25  (2.0, 4.0) | 3.25  (2.0, 5.0) | 3.75  (2.5, 5.2) | 5.75  (3.5, 9.0) |
| Fruits ^b^ | 0.5  (0.0, 1.25) | 1.0  (0.25, 2.0) | 1.5  (0.5, 3.0) | 2.0  (0.75, 4.0) | 3.0  (1.25, 7.5) |
| Seasonal fruits | 5.75  (2.5, 10.0) | 7.5  (3.5, 12.0) | 9.0  (5.0, 14.0) | 13.0  (7.5, 18.0) | 16.5  (10.0, 22.5) |
| Raw vegetables ^c^ | 1.75  (0.75, 3.0) | 2.0  (1.0, 4.0) | 3.0  (1.5, 5.0) | 3.25  (1.5, 7.0) | 5.5  (3.0, 10.0) |
| Sweet, cream & salted biscuits | 0.25  (0.0, 1.0) | 0.5  (0.0, 2.0) | 1.0  (0.25, 3.0) | 1.75  (0.0, 7.0) | 2.25  (0.25, 7.0) |
| High calories savoury snacks ^d^ | 0.75  (2.25, 1.5) | 1.0  (0.25, 1.5) | 1.25  (0.5, 2.25) | 1.5  (0.75, 3.0) | 2.0  (1.0, 3.75) |
| Ragi ball | 7.0  (0.5, 7.0) | 3.0  (0.0, 7.0) | 0.5  (0.0, 7.0) | 0.25  (0.0, 7.0) | 0.25  (0.0, 4.0) |
| Plain boiled white rice |  |  |  |  |  |
| **Rice- fried snacks-chicken-sweets** | | | | | |
| Tea & coffee | 3.0  (0.0, 14.0) | 7.0  (1.5, 14.0) | 8.0  (0.0, 14.0) | 14.0  (1.0, 14.0) | 14.0  (7.0, 21.0) |
| Fermented dairy products ^e^ | 1.0  (0.0, 3.1) | 1.0  (0.0, 3.5) | 2 .0  (0.5, 4.0) | 2.75  (1.0, 7.0) | 5.0  (1.0, 8.0) |
| Banana ^f^ | 2.0  (0.0, 4.0) | 3.0  (1.0, 7.0) | 3.0  (2.0, 7.0) | 4.0  (1.0, 7.0) | 7.0  (2.5, 7.0) |
| Plain boiled white rice | 7.0  (3.0, 14.0) | 14.0  (7.0, 14.0) | 14.0 (14.0,14.0) | 14.0  (14.0, 21.0) | 14.0  (14.0, 21.0) |
| Spice seasoned rice preparations ^g^ | 1.25  (0.5, 2.0) | 2.0  (1.0, 3.0) | 3.0 (2.0, 4.0) | 4.0  (2.0, 5.0) | 4.0  (3.0, 6.0) |
| Chicken ^h^ | 0.25  (0.0, 1.5) | 0.5  (0.0, 1.5) | 0.75  (0.0, 1.75) | 1.0  (0.25, 2.0) | 1.25  (0.5, 2.0) |
| Added sugar ^i^ | 8.5  (3.0, 15.5) | 14.0  (7.0, 17.0) | 14.0  (7.0, 19.0) | 15.0  (7.5, 21.0) | 16.0  (14.0, 26.1) |
| Medium calories savoury snacks ^j^ | 0.25  (0.0, 2.0) | 0.25  (0.0, 1.25) | 1.0  (0.0, 2.0) | 1.0  (0.0, 3.0) | 2.0  (0.5, 4.0) |
| Fast food ^k^ | 0.25  (0.0, 0.5) | 0.0  (0.0, 0.25) | 0.0  (0.0, 0.0) | 0.0  (0.0, 0.0) | 0.0  (0.0, 0.0) |
| Dried fruits ^l^ | 0.0  (0.0, 0.0) | 0.0  (0.0, 0.0) | 0.0  (0.0, 0.0) | 0.0  (0.0, 0.0) | 0.0  (0.0, 0.0) |
| **Healthy, traditional vegetarian** | | | | | |
| Fermented dairy products ^e^ | 1.0  (0.25, 2.5) | 1.0  (0.25, 4.0) | 1.5  (0.25, 4.0) | 3.0  (1.0, 7.0) | 6.0  (1.0, 8.0) |
| Ragi ball | 0.0  (0.0, 3.0) | 0.75  (0.0, 7.0) | 3.0  (0.0, 7.0) | 3.0  (0.0, 7.0) | 4.0  (0.5, 7.0) |
| Traditional whole and split pulse preparations | 4.0  (3.0, 7.5) | 4.0  (2.5, 7.0) | 5.0  (3.0, 7.5) | 7.0  (4.0, 9.0) | 6.5  (4.5, 10.5) |
| Traditional style GLV sides ^m^ | 2.25  (1.25, 4.0) | 3.0  (2.0, 4.0) | 3.0  (2.0, 4.0) | 3.0  (2.0, 4.5) | 3.5  (2.0, 5.0) |
| Raw vegetables ^c^ | 2.0  (1.0, 4.0) | 2.0  (1.0, 4.0) | 2.0  (1.0, 4.5) | 3.0  (1.5, 6.5) | 5.0  (2.2, 9.0) |
| Traditional, home-made sweets ^n^ | 0.5 (0.25,0.75) | 0.25  (0.0, 0.75) | 0.25  (0.0, 0.75) | 0.25  (0.0, 0.5) | 0.25  (0.0, 0.5) |
| Traditional shop bought sweets ^o^ | 0.0  (0.0, 0.0) | 0.0  (0.0, 0.0) | 0.0  (0.0, 1.0) | 0.0  (0.0, 1.0) | 0.0  (0.0, 1.0) |
| Fast food ^k^ | 0.0  (0.0, 0.5) | 0.0  (0.0, 0.0) | 0.0  (0.0, 0.0) | 0.0  (0.0, 0.0) | 0.0  (0.0, 0.25) |
| **Lighter shading indicates increasing consumption across quintiles, Darker shadings indicate decreasing consumption across quintiles**  ^a^ Other vegetables sides include ladys finger, gourds, pumpkin, capsicum, brinjal, beans, mushroom, multiveg saagu; ^b^ Fruits include all fruits except apple, yelakki banana, robusta banana, sweetlime, guava, pomegranate and green grapes; ^c^ Raw vegetables include cucumber, tomato, onion, carrot, radish, runner beans and raw greens; ^d^ High calories savoury snacks include pakoda/bonda/bhajji/samosa; ^e^ Fermented dairy products include buttermilk & raitha; ^f^ Banana include yelakki and robusta varieties; ^g^ Spice seasoned/flavoured rice preparations include tamarind, tomato, lemon, ghee, jeera, veg rice include pulao and bisibelebaath; ^h^ Chicken Preparations include fry and curry/ sambhar; ^I^ Added sugar include sugar in tea, coffee, milk, fruit juice, jam, honey and jaggery; ^j^ Medium calories savoury snacks include namkeen and chakli; ^k^ Fast foods include veg and non-veg pizza and burger; ^l^ Dried fruits inlcude figs, prunes and apricots; ^m^ Traditional style GLV sides include GLV sambhar, GLV palya and drumstick palya; ^n^ Traditional, home-made cereal based sweets such as kesaribaath, holige and kheer ; ^o^ Traditional shop bought fried sweets include khoa based sweets like barfi, pedha, jamoon, laddoo, halwa and other sweets. | | | | | |

**SUPPLEMENTARY SECTION 9**

**Figure (d): Correlations of the first five diet patterns derived by PCA of 205 foods and 68 food-groups (n=785)**


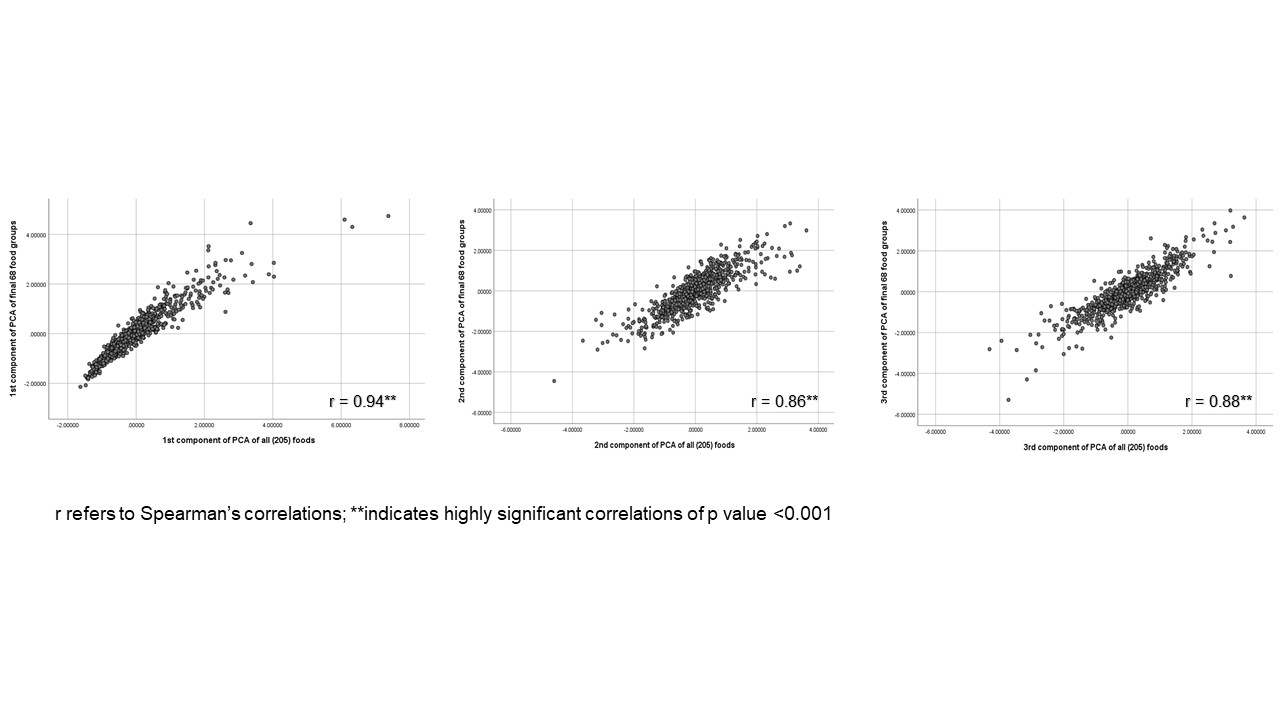

Supplement: Supplementary file 1 [file S1368980022001288sup.zip › S1368980022001288sup002.docx]
